# Method for the simultaneous isolation of primary astrocytes and microglia from the neonatal rats cerebral cortex

**DOI:** 10.3389/fncel.2026.1787397

**Published:** 2026-04-21

**Authors:** Kun Zhang, Songcan Wu, Yujie Zeng, Xinglin Wu, Meiming Qian, Kaya Xu

**Affiliations:** 1 College of Clinical Medicine, Guizhou Medical University, Guiyang, China; 2 Department of Neurosurgery, Affiliated Hospital of Guizhou Medical University, Guiyang, China

**Keywords:** astrocytes, graded serum, isolation, microglia, primary culture, time window

## Abstract

**Objective:**

To establish a protocol for the simultaneous isolation and high-purity purification of primary astrocytes and microglia from neonatal rats cerebral cortex.

**Methods:**

Single-cell suspensions were prepared from cerebral cortices of postnatal day 2 (P2) rat pups. Fibroblasts were pre-removed using differential adhesion techniques. Mixed glial cultures were maintained with graded serum (from 10% to 5% to 2% FBS) to suppress fibroblast proliferation. On day 14, microglia were isolated by constant temperature shaking (200 rpm, 12 h, 37 °C), followed by manual agitation of remaining adherent cells to purify astrocytes. Cell purity was assessed by immunofluorescence (Iba1 and GFAP) and validated by multicolor flow cytometry (CD11b/CD45; ACSA-2) and ER-TR7 fibroblast exclusion staining. Cell viability was evaluated by trypan blue exclusion and CCK-8 assay. Microglial morphology was quantified by cell body area, circularity index, and primary process number.

**Results:**

Day 14 was identified as the optimal separation time point. Immediately post-shaking, microglia purity (Iba1^+^) reached 98.6% ± 1.1%, and astrocyte purity (GFAP^+^) was 98.4% ± 1.7%. After subsequent purification culture, these values increased to 98.98% ± 1.21% and 98.81% ± 2.38%, respectively. Dual-label immunofluorescence confirmed minimal cross-contamination, with Iba1^+^/GFAP^+^ dual-positive cells constituting <1% in both populations. Multicolor flow cytometry corroborated these findings, yielding CD11b^+^ purity of 97.12% ± 1.58% for microglia (with 95.37% ± 1.84% classified as CD11b^+^/CD45^low homeostatic microglia) and ACSA-2^+^ purity of 94.65% ± 2.73% for astrocytes. No unequivocal ER-TR7^+^ fibroblasts were identified in either purified population. Microglial morphology progressively transitioned from amoeboid (Day 0: area 173.5 ± 32.8 μm^2^; circularity 0.847 ± 0.058; processes 0.8 ± 0.4) to ramified (Day 5: area 418.2 ± 68.3 μm^2^; circularity 0.438 ± 0.095; processes 4.3 ± 0.8 per cell). Cell viability remained above 92% following key procedural steps and recovered to over 95% post-purification; CCK-8 assay confirmed full metabolic recovery.

**Conclusion:**

This study establishes a combined method utilizing graded serum and constant temperature shaking for glial cell isolation, enabling simultaneous acquisition of both major glial cell types from a single animal. This cost-effective protocol provides a practical tool for functional studies of neuroglial cells.

## 1 Introduction

Astrocytes (AS) and microglia (MG) constitute the bulk of cells in the central nervous system (CNS), where they play crucial roles in maintaining neurovascular unit homeostasis and facilitating tissue repair after injury (Sofroniew, 2015). Recent studies indicate that bidirectional signaling between these two glial cell types plays a significant role in the pathogenesis of cerebral ischemia, epilepsy, and neurodegenerative diseases (Matejuk and Ransohoff, 2020; Vainchtein and Molofsky, 2020). Under ischemic conditions, activated microglia secrete inflammatory mediators, including IL-1α, TNF-α, and C1q, which can induce astrocytes to adopt a neurotoxic A1 phenotype, thereby exacerbating neuronal damage. Conversely, astrocytes can regulate microglial polarization through the release of exosomes or neurotrophic factors, thereby influencing the progression of neuroinflammation (Liddelow and Barres, 2017).

However, existing techniques for isolating primary glial cells struggle to balance high purity, cost-effectiveness, and the maintenance of optimal cellular states. Fibroblast contamination poses a particularly significant challenge in traditional co-culture systems, with reported rates reaching 10%–30%. This contamination not only dilutes the concentration of target cells but may also interfere with glial cell function via paracrine signaling (McCarthy and de Vellis, 1980; Stansley et al., 2012). Furthermore, the isolation process itself can induce cellular activation, leading to phenotypic alterations. These include the transition of astrocytes into a reactive state (Clarke et al., 2018) or the loss of resting characteristics in microglia (Nikodemova and Watters, 2012). A lack of standardized protocols across laboratories for critical parameters–such as animal age at harvest, enzymatic digestion conditions, and serum concentration–results in significant variability in cell yield and viability (Gingras et al., 2007; Grabert and McColl, 2018). More critically, although some protocols enable simultaneous isolation of multiple glial cell types (Leites and Morais, 2024; Samal et al., 2024), many commonly used methods still focus on isolating a single cell type per procedure (Bordt et al., 2020; Li and Zhang, 2024), which may increase overall animal usage when both cell types are required for comparative studies. This not only increases experimental costs and time but also contradicts the core principle of “Reduction” outlined in the ethical “3Rs” framework for laboratory animal use.

Magnetic-activated cell sorting (MACS) and flow cytometric cell sorting (FACS) technologies enable the efficient enrichment of target cells through the use of specific antibody labeling. Related studies demonstrate that these methods can achieve nearly 99% purity in isolated cell populations (Pan and Wan, 2020; Zelenka et al., 2022). However, the high cost per isolation and requirement for specialized equipment limit their adoption in routine laboratories (Bohlen et al., 2019). More critically, studies indicate that antibody binding and fluid shear stress during flow sorting may induce non-specific activation of microglia, altering their resting transcriptional profiles (Ocañas et al., 2022). An in-depth analysis of this intercellular communication mechanism requires the establishment of high-purity, high-activity *in vitro* co-culture models. In recent years, multicellular co-culture systems have demonstrated significant application value in neurodegenerative disease research (Luchena et al., 2022; Hwang et al., 2022), while the development of humanized iPSC platforms has provided new avenues for translational research (Tujula et al., 2025). In contrast, traditional shaker-based methods remain widely adopted due to their simplicity and low cost, though improvements in cell purity and state maintenance are still needed.

To address these limitations, this study systematically optimized pre-processing parameters–including harvest age, enzymatic digestion conditions, and substrate coating–to establish a synchronized method for primary glial cell isolation based on serum gradient concentration regulation and optimization of the separation time window. The selection of the isolation time window was based on classical literature reports. Tamashiro et al. (2012) demonstrated that mixed glial cultures typically form stable stratified structures between days 7 and 21. Preliminary experiments indicated that the stratified structure was immature on day 7, whereas astrocytes became excessively confluent after day 17. Therefore, days 7, 10, 14, and 17 were selected as representative time points for systematic comparison. A three-step purification process–consisting of differential adhesion, gradient serum culture, and modified oscillatory separation–was employed to simultaneously isolate highly purified astrocytes and microglia from the cerebral cortices of newborn Sprague-Dawley (SD) rat pups. Through systematic optimization of harvest age, enzymatic digestion conditions, matrix coating, and culture parameters, a three-step purification workflow was established, based on differential adhesion, gradient serum selection, and modified oscillatory separation.

The key technical aspects of this protocol were as follows: (1) selection of postnatal day 2 (P2) rat pups, involving meticulous dura mater dissection followed by mild papain digestion and poly-D-lysine (PDL) substrate coating; (2) implementation of a 10% → 5% → 2% serum gradient to inhibit fibroblast proliferation while supporting glial cell growth; (3) identification of days 10–14 as the optimal isolation window, with day 14 representing the peak time point; and (4) simultaneous isolation of both glial cell types from a single animal, thereby reducing overall animal use. As this method requires only standard cell culture equipment, it significantly reduces costs compared to immunological separation techniques. Thus, it provides a practical tool for studying glial cell interaction mechanisms, neuroinflammation, and neurodegenerative diseases.

## 2 Materials and methods

### 2.1 Experimental materials

This study utilized SPF-grade Sprague-Dawley (SD) newborn rats at postnatal day 2 (P2), which were procured from Suzhou Xishan Biotechnology Co., Ltd., (Laboratory Animal Production License No.: SCXK (Jing) 2024-0003). All animal experimental protocols were approved by the Animal Ethics Committee of Guizhou Medical University (Approval No. 2502945). The key reagents and equipment used in this study are listed in Table 1.

**TABLE 1:** Main reagents and equipment used in the experiment.

Reagent or resource	Source	Identifier
Antibodies		
Rabbit anti-Iba1 antibody	Abcam, UK	Cat.#ab178846; RRID:AB_2636859
Rabbit anti-GFAP antibody	Abcam, UK	Cat.#ab7260; RRID:AB_305808
Goat anti-rabbit IgG H&L (Alexa Fluor 488)	Abcam, UK	Cat.#ab150077; RRID:AB_2630356
Cy3-conjugated goat anti-rabbit IgG	Beyotime biotechnology, Shanghai, China	Cat.#A0516
Mouse anti-GFAP (clone GA5)	Cell signaling technology, USA	Cat.#3670
Goat anti-rabbit IgG AF488, highly cross-adsorbed	Invitrogen, USA	Cat.#A-11034
Goat anti-mouse IgG AF594, highly cross-adsorbed	Invitrogen, USA	Cat.#A-11032
Rat anti-ER-TR7	Thermo Fisher Scientific, USA	Cat.#MA1-40076
Goat anti-rat IgG AF594, cross-adsorbed	Invitrogen, USA	Cat.#A-11007
PE anti-CD11b (clone M1/70)	BioLegend, USA	Cat.#101208
FITC anti-CD45 (clone 30-F11)	BioLegend, USA	Cat.#103108
ACSA-2-APC (clone IH3-18A3)	Miltenyi Biotec, Germany	Cat.#130-117-535
Zombie NIR fixable viability kit	BioLegend, USA	Cat.#423105
Purified anti-CD16/CD32 (clone 2.4G2)	BD Biosciences, USA	Cat.#553142
Chemicals, peptides, and recombinant proteins		
Papain	Solarbio, Beijing, China	Cat.#G8430
Penicillin-streptomycin (100×)	Sigma-Aldrich, USA	Cat.#V900929
Poly-D-Lysine (PDL)	Sigma-Aldrich, USA	Cat.#P6407
DMEM, high glucose	Thermo Fisher Scientific, USA	Cat.#1965092
Fetal bovine serum (FBS)	Thermo Fisher Scientific, USA	Cat.#10100147C
Normal goat serum	Solarbio, Beijing, China	Cat.#SL038
Triton X-100	Solarbio, Beijing, China	Cat.#T8200
Paraformaldehyde (PFA)	Sigma-Aldrich, USA	CAS: 30525-89-4
DAPI mounting medium (Antifade)	Solarbio, Beijing, China	Cat.#S2110
Trypsin-EDTA (0.25%)	Gibco/Thermo Fisher Scientific, USA	Cat.#25200056
Phosphate Buffered Saline (PBS), pH 7.4	Gibco/Thermo Fisher Scientific, USA	Cat.#10010023
Poly-L-Lysine (PLL)	Sigma-Aldrich, USA	Cat.#P4707
CCK-8 (Cell Counting Kit-8)	Dojindo, Japan	Cat.#CK04
Staurosporine	Sigma-Aldrich, USA	Cat.#S5921
ProLong Diamond Antifade Mountant	Invitrogen, USA	Cat.#P36961
Equipment		
Microdissection instruments	RWD Life Science, Shenzhen, China	N/A
Axioskop 40 upright fluorescence microscope	Carl Zeiss, Germany	N/A
TE2000-U inverted fluorescence microscope	Nikon, Japan	N/A
MCO-170-SR CO_2_ incubator	Bingshan Songyang Biotech, Dalian, China	N/A
T75 cell culture flasks	Corning/Thermo Fisher Scientific, USA	Cat.#430641U
6-well cell culture plates	Corning/Thermo Fisher Scientific, USA	Cat.#3516
200-mesh nylon cell strainer (74 μm)	BD Falcon, USA	Cat.#352350
Horizontal orbital shaker with temperature control	Kylin-Bell Lab Instruments, China	N/A
BD FACSCanto II flow cytometer	BD Biosciences, USA	N/A
Multiskan FC microplate reader	Thermo Fisher Scientific, USA	N/A
Other		
NIH 3T3 mouse embryonic fibroblasts	ATCC, USA	CRL-1658

### 2.2 Mixed glial cell culture

**FIGURE 1:**
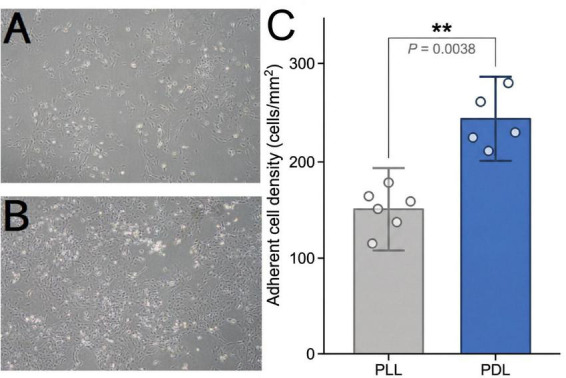
Effects of matrix types on primary mixed glial cells. **(A)** PLL-coated group at Day 3. **(B)** PDL-coated group at Day 3. Scale bar = 100 μm. **(C)** Quantitative comparison of adherent cell density between PLL- and PDL-coated groups. Data are presented as mean ± SD with individual data points (*n* = 5). **P < 0.01, unpaired Student’s *t*-test.

Two candidate substrates, poly-L-lysine (PLL) and poly-D-lysine (PDL), were compared. T75 culture flasks were coated with 0.1 mg/mL PLL or PDL working solution. After incubation at 37 °C for 2 h, the coating solution was aspirated, and the flasks were rinsed twice with phosphate-buffered saline (PBS) before use. A preliminary assessment after 3 days in culture indicated that the PDL-coated group exhibited significantly higher adherent cell density and more uniform cell distribution compared to the PLL-coated group (see Results, Section “3.1 Effects of Coating Materials on Cell Morphology”). Adherent cell density was quantified on day 3 post-seeding by selecting five non-overlapping fields of view (100× magnification) per independent experiment and counting cells using ImageJ software. PDL-coated flasks yielded a significantly higher cell density (245.8 ± 38.4 cells/mm^2^) compared with PLL-coated flasks [152.6 ± 41.7 cells/mm^2^; t(8) = 3.734, *P* = 0.0038, unpaired Student’s *t*-test; Cohen’s *d* = 2.33; Figure 1C]. The inter-experiment coefficient of variation (CV) was also lower in the PDL group (15.6%) than in the PLL group (27.3%), indicating superior batch-to-batch reproducibility. Therefore, PDL was selected as the coating material for all subsequent experiments.

Inside a UV-sterilized (30 min) ultra-clean workbench, three 6-cm culture dishes were prepared, each filled with an equal volume of DMEM medium. The dishes were then placed on an ice tray for pre-cooling. Postnatal day 2 (P2) newborn rats were disinfected with 75% ethanol and placed inside the ultra-clean workbench. Following anesthesia, rats were euthanized by cervical dislocation and immediately decapitated. The heads were then rinsed 2–3 times in ice-cold (4 °C) DMEM to remove residual blood. Each head was transferred to a prepared Petri dish (designated as Dish 1), secured with straight forceps, and the scalp was carefully peeled away using curved forceps. The skull was incised along the sagittal suture, and the bone layers were gently dissected away layer by layer. Curved forceps were positioned beneath the foramen magnum, and the entire brain tissue was completely extracted with a gentle rotational and lifting motion. All dissection steps were performed on an ice-cold surface maintained at 0 °C–4 °C.

The brain tissue was transferred to Petri dish #2 containing pre-chilled (4 °C) DMEM. Under a dissecting microscope, fine-tipped straight forceps were used to carefully peel the pia mater and associated blood vessels from the cerebral cortex surface. The cortex was then divided into 1–2 mm^3^ chunks using curved forceps and transferred to Petri dish #3. The medium was aspirated, 10 mL of 0.25% papain (pre-warmed to 37 °C) was added, and the tissue was incubated at 37 °C for 20 min. The dish was gently shaken 2–3 times at 7–8 min intervals (Hu et al., 2022). Enzymatic digestion was terminated by adding an equal volume of serum-containing complete medium. The tissue chunks were gently triturated approximately 20 times using a 1 mL pipette tip to obtain a uniform single-cell suspension, with care taken to avoid bubble formation. The suspension was filtered through a 70 μm cell strainer into a 50 mL centrifuge tube. The supernatant was discarded and the cell pellet was resuspended in complete medium. To remove adhering fibroblasts, the cell suspension was transferred to an uncoated culture dish and incubated at 37 °C for 15 min. Finally, the supernatant containing the non-adherent cells was collected and seeded into a poly-D-lysine (PDL)-precoated T75 flask. The cells were cultured at 37 °C in a humidified incubator with 5% CO_2_.

### 2.3 Isolation and purification of primary microglia and astrocytes

Mixed glial cells were cultured following a graded serum protocol. The cells were initially maintained in complete DMEM medium containing 10% FBS from day 1 to day 3. The medium was then changed to contain 5% FBS from day 4 to day 7, and subsequently to 2% FBS from day 8 to day 14, to continuously suppress fibroblast proliferation. During the culture period, the medium was changed every 2–3 days, with approximately two-thirds of the volume replaced each time. On day 14, the culture flasks were sealed and incubated on a constant-temperature horizontal shaker at 200 rpm and 37 °C for 12 h (Tamashiro et al., 2012). After the shaking step, the supernatant was collected and centrifuged at 1000 rpm for 3 min. The resulting supernatant was discarded, and the cell pellet was resuspended in complete medium containing 10% FBS. The resuspended cells were then seeded into poly-D-lysine (PDL)-coated 6-well plates or culture flasks. After 5 days of culture, Iba1 immunofluorescence staining was performed to identify the microglia and quantify their purity.

**FIGURE 2:**
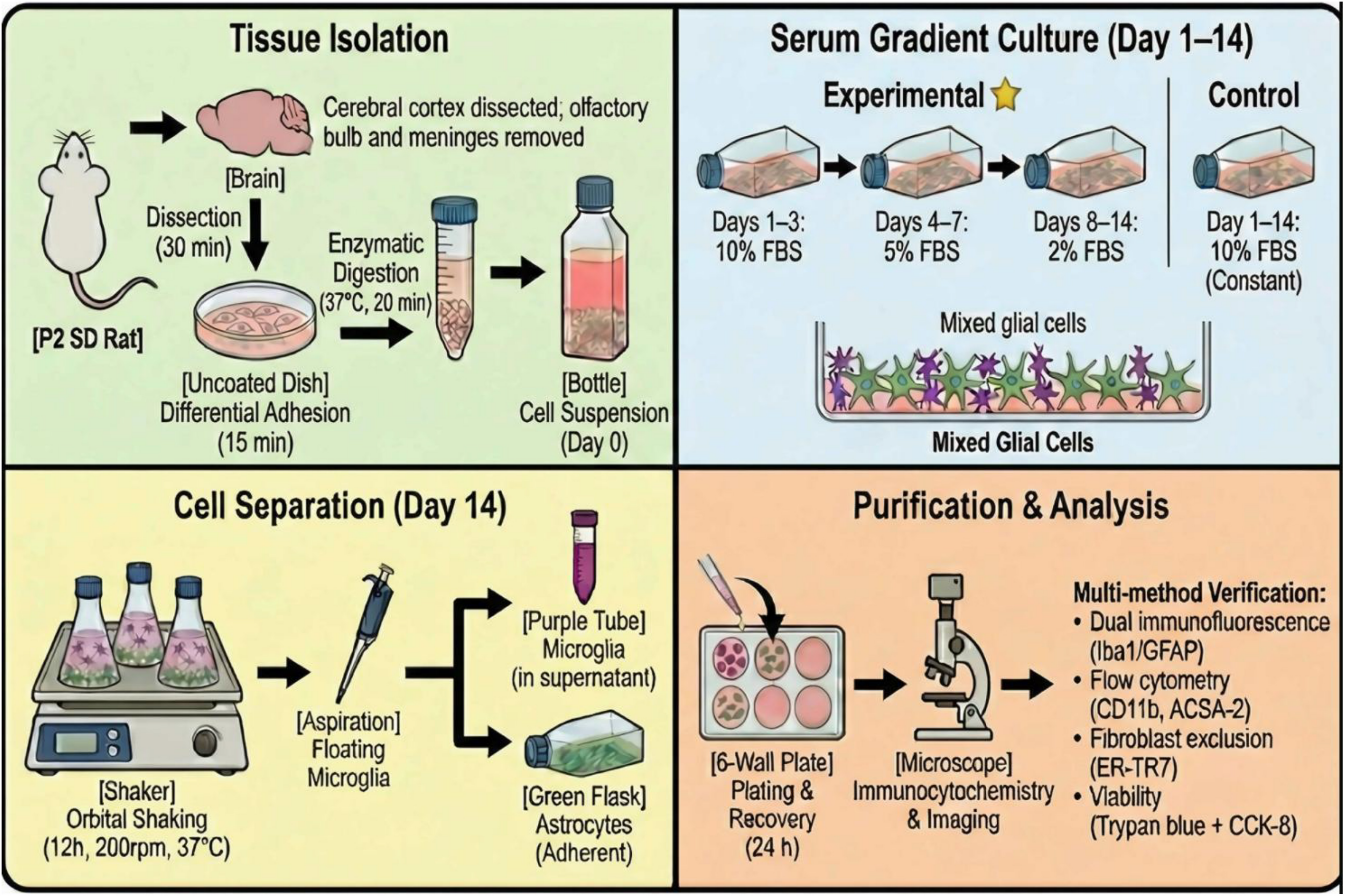
Schematic diagram of primary microglia and astrocyte isolation and purification protocol.

To the original culture flask (which remained after the shaking separation step), 10 mL of fresh DMEM medium was added for continued culture. During each medium change, a cross-hand shaking method was applied (30 oscillations each in the front-to-back and left-to-right directions, lasting 5 min) to remove residual non-astrocytic cells. When the cells reached 70%–80% confluence, they were digested with 0.25% trypsin-EDTA solution for 3–5 min. The digestion was terminated by adding serum-containing complete medium, and the cells were then passaged at a 1:3 ratio into new 6-well plates. Upon reaching complete confluence, GFAP immunofluorescence staining was performed to assess astrocyte purity. A simplified representation of the experimental procedure described above is presented in Figure 2.

To determine the optimal separation time window, shaking separation experiments were conducted on days 7, 10, 14, and 17 of the mixed glial cell culture. Five independent experiments were performed at each time point, with one P2 newborn rats used per experiment (*n* = 5 per time point). The procedure at each time point mirrored that described for day 14: following oscillatory separation, the isolated microglia were subcultured for 5 days, whereas the remaining adherent astrocytes were maintained in culture until confluence. Subsequently, Iba1 and GFAP immunofluorescence staining were performed to quantitatively assess the purity of the respective cell populations.

### 2.4 Gradient serum vs. constant serum control experiment

To validate the superiority of the gradient serum strategy over the traditional constant-serum culture, we performed a systematic control experiment. The experiment consisted of two groups: the experimental group followed the gradient serum strategy (using DMEM complete medium with 10% FBS on days 1–3, switching to medium with 5% FBS on days 4–7, and maintaining 2% FBS on days 8–14), while the control group was cultured continuously in DMEM complete medium containing 10% FBS throughout the entire period (days 1–14). All other culture conditions (PDL coating, differential adhesion, and shaking parameters) were identical between the groups (see Methods, sections 2.2–2.3).

### 2.5 Cell density and viability assay

#### 2.5.1 Cell density quantification and trypan blue exclusion assay

Total cells were counted using a hemocytometer to calculate the seeding density. At 24 h post-seeding, cells were observed and photographed under an inverted microscope. Five non-overlapping fields of view were randomly selected (at 100× magnification), and the number of cells in each field was counted using ImageJ software. The cell density per unit area (cells/cm^2^) was calculated based on the field area (0.785 mm^2^ per field, 1 mm in diameter). The average of the five fields was taken as the cell density for that sample.

Cell viability was assessed following critical procedural steps using the trypan blue exclusion assay. A 100 μL aliquot of the cell suspension was mixed with an equal volume of 0.4% trypan blue solution. After gentle pipetting 3–5 times to ensure thorough mixing, the mixture was incubated at room temperature for 2–3 min. Subsequently, 10 μL of the mixture was loaded into a Neubauer hemocytometer. Viable cells (unstained and refractile) and non-viable cells (blue-stained) were counted separately under an inverted phase-contrast microscope at 100× magnification. For each sample, cells were counted in the four corner quadrants of the hemocytometer. A minimum of 20 cells was counted per large square (1 mm^2^) in each quadrant. Cell viability was calculated using the following formula: Viability (%) = [Number of Viable Cells/(Number of Viable Cells + Number of Non-viable Cells)] × 100.

Survival rate was assessed at the following critical time points: after papain digestion (T0), after differential adhesion (T1), after shaking separation on day 14 (T2), on day 5 of the glial cell purification culture (T3), and on day 3 after astrocyte passaging (T4). Each time point was assessed in triplicate, with two technical replicates per biological replicate. Data are presented as mean ± standard deviation (Mean ± SD).

#### 2.5.2 CCK-8 metabolic viability assay

To complement the membrane-integrity-based assessment provided by trypan blue exclusion, metabolic viability was independently evaluated using the Cell Counting Kit-8 (CCK-8; Dojindo, CK04), which measures mitochondrial dehydrogenase activity. Purified microglia or astrocytes were seeded at 1.5 × 10^4^ cells/well in PDL-coated 96-well plates containing 100 μL phenol-red-free DMEM supplemented with 10% FBS. CCK-8 assays were performed at the same time points as the trypan blue assay (T0–T4). For T0 and T1 samples, cells were allowed to adhere for 24 h before measurement; T2–T4 samples were measured directly at the corresponding culture stages.

At each time point, 10 μL of CCK-8 reagent was added per well, and the plates were incubated at 37 °C in 5% CO_2_ for 2.5 h (astrocytes) or 3.5 h (microglia). The incubation durations were empirically optimized for each cell type to ensure that absorbance values fell within the linear detection range of the assay; the longer incubation for microglia was necessitated by their comparatively lower mitochondrial dehydrogenase activity relative to astrocytes under the culture conditions employed. Absorbance was measured at 450 nm with a reference wavelength of 650 nm using a Multiskan FC microplate reader (Thermo Fisher Scientific). Each experiment included the following controls: medium-only blank wells (background subtraction), 1 μM staurosporine-treated wells (24 h; death control; Sigma-Aldrich, S5921), and untreated cell wells (100% viability reference). Six technical replicate wells were included per condition, and five independent biological replicates were performed (*n* = 5). Metabolic viability was calculated as: Metabolic viability (%) = [(OD_sample − OD_blank)/(OD_untreated − OD_blank)] × 100%.

### 2.6 Immunofluorescence Staining

#### 2.6.1 Single-label immunofluorescence staining

Single-label immunofluorescence was employed for rapid purity assessment at each time-window screening stage. Cells were seeded on glass coverslips pre-positioned within 6-well plates and maintained in culture until attaining appropriate confluency. Cells were washed three times with ice-cold PBS (pH 7.4) for 3 min per wash. Cells were then fixed with 4% paraformaldehyde (PFA) at room temperature for 20 min, followed by three washes with PBS. For permeabilization, the cells were incubated with 0.25% Triton X-100 at room temperature for 15 min, followed by three PBS washes. Next, the cells were blocked by incubation with 10% normal goat serum at room temperature for 30 min. After removal of the blocking solution, the cells were incubated overnight (12–16 h) at 4 °C with the appropriate primary antibody diluted in blocking buffer: either rabbit anti-GFAP antibody (1:200) for astrocyte identification or rabbit anti-Iba1 antibody (1:200) for microglia identification.

The next day, coverslips were retrieved, allowed to equilibrate to room temperature for 30 min, and then washed three times with PBS. Cells were then incubated for 1 h at room temperature in the dark with the corresponding fluorescently labeled secondary antibody: either Alexa Fluor 488-conjugated goat anti-rabbit IgG (1:200) for GFAP detection or Cy3-conjugated goat anti-rabbit IgG (1:200) for Iba1 detection. After three additional PBS washes, nuclei were stained by incubating the cells with DAPI working solution (diluted 1:1000) at room temperature in the dark for 5 min. The cells were then rinsed twice with PBS. Finally, the coverslips were mounted onto glass slides using an anti-fade mounting medium. Each coverslip was placed cell-side down onto a slide and gently pressed to remove any air bubbles. The mounted samples were then observed under a fluorescence microscope.

#### 2.6.2 Dual-label immunofluorescence staining

**FIGURE 3:**
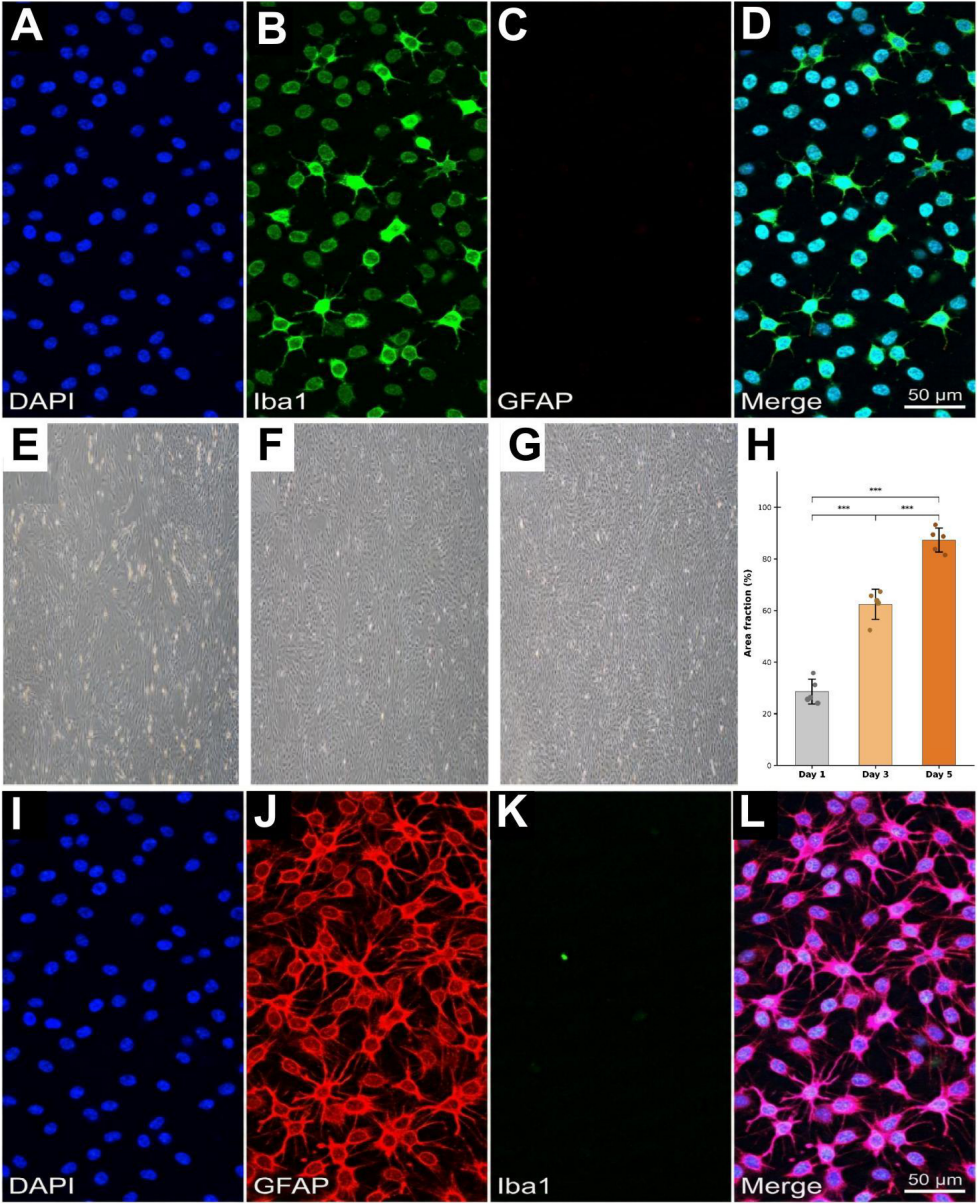
Immunofluorescence characterization of purified microglia and astrocytes. **(A–D)** Dual-label immunofluorescence identification of purified microglia at Day 5 post-isolation. **(A)** DAPI nuclear staining (blue). **(B)** Iba1 immunostaining (green, Alexa Fluor 488). **(C)** GFAP immunostaining (red, Alexa Fluor 594). **(D)** Merged image. Scale bar = 50 μm. Magnification: 200×. **(E–H)** Phase contrast microscopy of purified astrocytes during recovery culture. **(E)** Day 1 post-passage. **(F)** Day 3 post-passage. **(G)** Day 5 post-passage showing confluent monolayer with typical “cobblestone” morphology. **(H)** Quantification of astrocyte area fraction over culture days (Mean ± SD, *n* = 5). ***P < 0.001 (one-way ANOVA with Tukey’s *post-hoc* test). **(I–L)** Dual-label immunofluorescence identification of purified astrocytes at Day 5 post-passage. **(I)** DAPI nuclear staining (blue). **(J)** GFAP immunostaining (red, Alexa Fluor 594). **(K)** Iba1 immunostaining (green, Alexa Fluor 488). **(L)** Merged image. Scale bar = 50 μm. Magnification: 200×.

To simultaneously detect astrocyte and microglial markers within the same field of view, dual-label immunofluorescence was performed on the final purified cell populations (Figures 3A–D, I–L). Iba1 and GFAP were selected as microglial and astrocyte markers, respectively, as they represent the most widely adopted immunocytochemical markers for cell-type identification in primary glial cultures and enable direct comparison with previously published isolation protocols (Tamashiro et al., 2012; Zelenka et al., 2022). To enable species-specific secondary antibody discrimination, the dual-label protocol paired rabbit anti-Iba1 (Abcam, ab178846; 1:500) with mouse anti-GFAP (Cell Signaling Technology, #3670, clone GA5; 1:500), replacing the rabbit anti-GFAP used in single-label staining.

Cells on glass coverslips were processed as described in Section “2.6.1 Single-label immunofluorescence staining,” with the following modifications: the Triton X-100 concentration was reduced to 0.1% and the blocking duration was extended to 1 h. Coverslips were then co-incubated overnight at 4 °C with both primary antibodies diluted together in antibody diluent.

The following day, coverslips were washed three times with PBS and co-incubated for 1 h at room temperature in the dark with two highly cross-adsorbed secondary antibodies: goat anti-rabbit IgG Alexa Fluor 488 (Invitrogen, A-11034; 1:500) and goat anti-mouse IgG Alexa Fluor 594 (Invitrogen, A-11032; 1:500). After PBS washes, nuclei were counterstained with DAPI (1 μg/mL) for 5 min and coverslips were mounted with ProLong Diamond Antifade Mountant (Invitrogen, P36961). Images were acquired using an Axioskop 40 upright fluorescence microscope at 20× magnification; five non-overlapping fields were randomly captured per condition, with a minimum of 300 DAPI^+^ cells counted per condition.

Negative controls included: (i) secondary-antibody-only controls (primary antibodies omitted); (ii) Iba1-only single-stain controls; (iii) GFAP-only single-stain controls; and (iv) pre-separation mixed glial cultures as a positive control. Cells in each field were classified into four categories: Iba1^+^/GFAP^–^ (microglia), Iba1^–^/GFAP^+^ (astrocytes), Iba1^+^/GFAP^+^ (dual-positive), and Iba1^–^/GFAP^–^ (unlabeled).

### 2.7 Flow cytometry

To provide an independent, quantitative validation of cell purity complementary to immunofluorescence, multicolor flow cytometry was performed on purified microglia (day 5 of purification culture) and astrocytes (day 3 post-passage) separately.

Cell preparation: adherent cells were dissociated with 0.25% trypsin-EDTA (37 °C, 3–5 min), neutralized with serum-containing complete medium, gently triturated, and sequentially filtered through 70 μm and 35 μm cell strainers to obtain single-cell suspensions. Cells were washed with FACS buffer (PBS containing 2% FBS and 2 mM EDTA) and adjusted to 2 × 10^5^ cells per tube.

Fc receptor blocking: to minimize non-specific Fc receptor binding, cells were incubated with purified anti-CD16/CD32 (BD Biosciences, 553142, clone 2.4G2) on ice for 15 min.

Surface marker staining: the following antibody panel was added and incubated on ice in the dark for 25 min: PE anti-CD11b (BioLegend, 101208, clone M1/70), FITC anti-CD45 (BioLegend, 103108, clone 30-F11), ACSA-2-APC (Miltenyi Biotec, 130-117-535, clone IH3-18A3), and Zombie NIR fixable viability dye (BioLegend, 423105). Cells were washed twice with FACS buffer and resuspended for acquisition.

Data acquisition and analysis: data were acquired on a BD FACSCanto II flow cytometer, recording ≥20,000 live-cell events per sample. The gating strategy was as follows: FSC-A/SSC-A scatter plot to exclude debris → FSC-H/FSC-A to exclude doublets → Zombie NIR→ gate to select live cells → marker-specific analysis. Positive thresholds for each channel were set using fluorescence-minus-one (FMO) controls. Data were analyzed with FlowJo software (v10.8, BD Biosciences). Microglia were defined as CD11b^+^/CD45low cells; astrocytes were defined as ACSA-2^+^ cells. Five independent experiments were performed (*n* = 5).

### 2.8 Fibroblast exclusion assay

To confirm the effective removal of fibroblasts by the gradient serum starvation and differential adhesion steps, immunofluorescence co-staining for the reticular fiber marker ER-TR7 was performed on purified cell populations. ER-TR7 was selected in preference to CD90/Thy1 on the basis of marker specificity within the neural cell context. ER-TR7 specifically recognizes reticular fibroblasts, including those of meningeal origin (Van Vliet et al., 1986; Schiavinato et al., 2021), which represent the predominant contaminating cell type in neonatal brain-derived primary glial cultures (Tamashiro et al., 2012). By contrast, CD90 (Thy1) exhibits broad expression across multiple cell lineages in rodent central nervous system tissue–including neurons, T-lymphocyte subsets, and certain mesenchymal progenitor populations–thereby precluding its reliable use as a fibroblast-specific exclusion marker in preparations containing heterogeneous neural cell types.

Purified astrocytes and microglia were seeded onto PDL-coated glass coverslips in 6-well plates and cultured until appropriate confluency. Fixation and permeabilization procedures were identical to those described in Section “2.6.2 Dual-label immunofluorescence staining.” After blocking with 10% normal goat serum for 1 h, coverslips were co-incubated overnight at 4 °C with rat anti-ER-TR7 (Thermo Fisher Scientific, MA1-40076; 1:30) together with either rabbit anti-GFAP (Abcam, ab7260; 1:1000) or rabbit anti-Iba1 (Abcam, ab178846; 1:500).

The following day, coverslips were washed with PBS and incubated for 1 h at room temperature in the dark with goat anti-rat IgG Alexa Fluor 594 (Invitrogen, A-11007, cross-adsorbed; 1:500) and goat anti-rabbit IgG Alexa Fluor 488 (Abcam, ab150077; 1:500). Nuclei were counterstained with DAPI and coverslips were mounted with ProLong Diamond.

NIH 3T3 mouse embryonic fibroblasts (ATCC CRL-1658) served as a positive control for ER-TR7 staining. For each condition, five non-overlapping fields were randomly captured and a minimum of 200 DAPI^+^ cells were counted. The ER-TR7 positivity threshold was determined with reference to the mean fluorescence intensity of the NIH 3T3 positive control.

### 2.9 Morphological quantification

**FIGURE 4:**
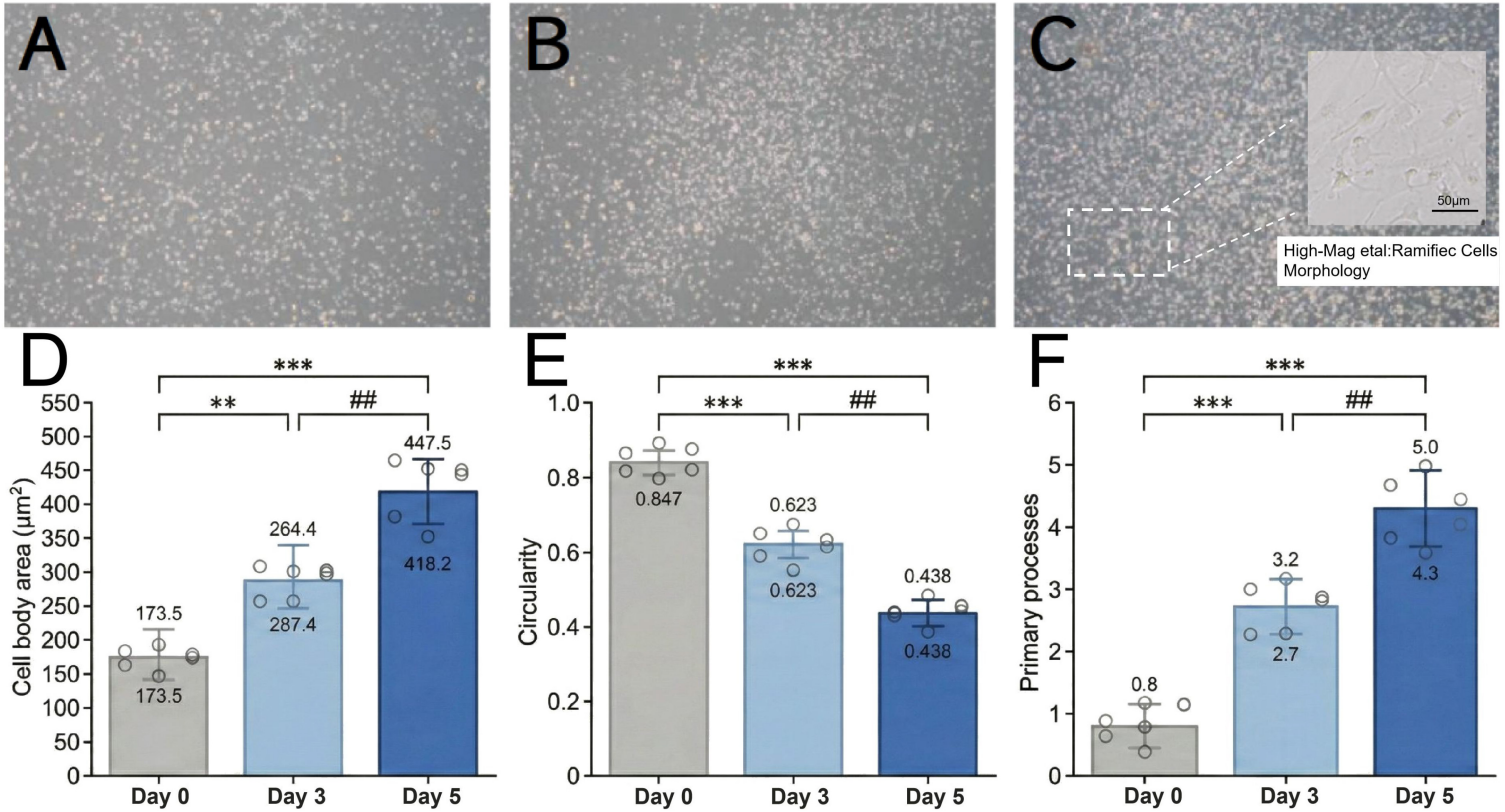
Changes in microglial morphology over culture time. **(A)** Day 0 (day of isolation and purification). **(B)** Day 3 of culture. **(C)** Day 5 of culture. All images at 100× magnification. Scale bar = 100 μm. **(D)** Cell body area (μm^2^). **(E)** Circularity index. **(F)** Number of primary processes. Data are presented as mean ± SD with individual data points (*n* = 5). **P < 0.01, ***P < 0.001 vs. Day 0; ##*P* < 0.01 vs. Day 3 (one-way ANOVA with Tukey’s *post-hoc* test).

Microglial morphology (Figures 4D–F): Using ImageJ software, individual microglial cell bodies were manually delineated on phase-contrast images (100× magnification) using the Freehand Selection tool, followed by the Measure command to obtain the following parameters: (i) cell body area (μm^2^); (ii) circularity index (4π × area/perimeter^2^, range 0–1, where 1 represents a perfect circle). Primary process number was determined by manual counting of processes emanating directly from the soma. A minimum of 30 cells per condition per experiment were analyzed.

Although advanced morphometric methods–including Sholl analysis, automated branching quantification, and dedicated plugins such as GliaMorph and 3DMorph–enable detailed characterization of microglial arbor complexity, their application requires high-resolution confocal images of fluorescently labeled cells with individually resolved dendritic arbors at low plating density. In the present study, morphological monitoring was conducted using phase-contrast microscopy (100× magnification) on cultures maintained at moderate-to-high confluency, conditions under which the optical contrast and spatial resolution are insufficient for reliable automated arbor reconstruction. Accordingly, cell body area, circularity index, and primary process number were adopted as morphometric parameters amenable to reproducible quantification from phase-contrast micrographs using standard ImageJ measurement tools.

Astrocyte spreading area (Figure 3H): GFAP immunofluorescence images were binarized using the Threshold function in ImageJ (Otsu auto-threshold method), and the Area Fraction (%) was obtained via Analyze ^→^ Measure. Five non-overlapping fields at 20× magnification were randomly selected per condition per experiment.

### 2.10 Image acquisition and quantitative data analysis

Unless otherwise specified, five independent experiments were performed for each assay (*n* = 5), with one P2 newborn rat used per experiment. Fluorescent images were acquired using either an Axioskop 40 upright fluorescence microscope (Carl Zeiss, Germany) or a TE2000-U inverted fluorescence microscope (Nikon, Japan). Specific image acquisition parameters, including field number, magnification, and minimum cell counts, are detailed in the corresponding subsections (Sections “2.6.1 Single-label immunofluorescence staining” and “2.6.2 Dual-label immunofluorescence staining”). All images within each experiment were captured under identical exposure settings.

Quantitative analysis of cell purity was conducted using ImageJ software (NIH, version 1.53). The total number of cell nuclei was counted from the DAPI channel, while positively labeled cells were counted from the specific marker channel (GFAP or Iba1). Cell purity was calculated using the following formula: Purity (%) = (Number of positively labeled cells/Total number of DAPI^+^ nuclei) × 100. Purity was quantified based on DAPI^+^ nuclear counts, which remain individually resolvable in confluent monolayers irrespective of cell-body overlap. Data obtained from the 4 to 6 fields per experiment were pooled and averaged.

For the cell density and viability assays, each sample was analyzed in duplicate (two technical replicates), and the mean value was calculated. All statistical analyses were performed using GraphPad Prism software (version 9.0). For comparisons between two groups (e.g., PDL vs. PLL coating, graded serum reduction vs. constant serum culture), an unpaired Student’s *t*-test was employed. For comparisons among three or more groups (e.g., purity and viability at different time points), one-way analysis of variance (ANOVA) was performed. When the ANOVA indicated a significant difference, Tukey’s honest significant difference (HSD) *post-hoc* test was applied for multiple comparisons. Trypan blue and CCK-8 viability data obtained at identical time points were compared using a paired-sample *t*-test. Effect sizes are reported as Cohen’s d. The significance levels were defined as follows: *P < 0.05, **P < 0.01, and ***P < 0.001; “ns” denotes not significant (*P* > 0.05). Data are presented as the mean ± standard deviation (SD). The normality of data distribution was assessed using the Shapiro-Wilk test, and the homogeneity of variances was tested using Levene’s test.

## 3 Results

### 3.1 Effects of coating materials on cell morphology

As detailed in Section “2.2 Mixed glial cell culture,” the effects of poly-L-lysine (PLL) and poly-D-lysine (PDL) as coating substrates on cell adhesion and morphology were compared. Phase-contrast microscopy revealed distinct morphological differences between cells grown on the two substrates (Figure 1). In the PLL-coated group (Figure 1A), cells exhibited a scattered distribution with relatively low density. Some cells appeared rounded with underdeveloped processes, and a subset demonstrated unstable adhesion during routine medium changes.

In contrast, the PDL-coated group (Figure 1B) displayed higher cell density and a more uniform distribution. The majority of cells exhibited well-developed spreading. Astrocytes exhibited a characteristic stellate morphology with abundant processes. Microglia displayed a branched morphology, forming intercellular networks.

Quantitative analysis confirmed these observations: PDL-coated flasks yielded a significantly higher adherent cell density (245.8 ± 38.4 cells/mm^2^) compared with PLL-coated flasks (152.6 ± 41.7 cells/mm^2^; *P* = 0.0038, unpaired Student’s *t*-test, Cohen’s *d* = 2.33; Figure 1C), representing an approximately 61% increase. The inter-field coefficient of variation was also lower in the PDL group (15.6%) than in the PLL group (27.3%), indicating more uniform cell distribution. Although both substrates supported primary cell culture, PDL was selected for all subsequent experiments based on its significantly superior cell adhesion and distribution properties

### 3.2 Determination of the optimal separation time window

**FIGURE 5:**
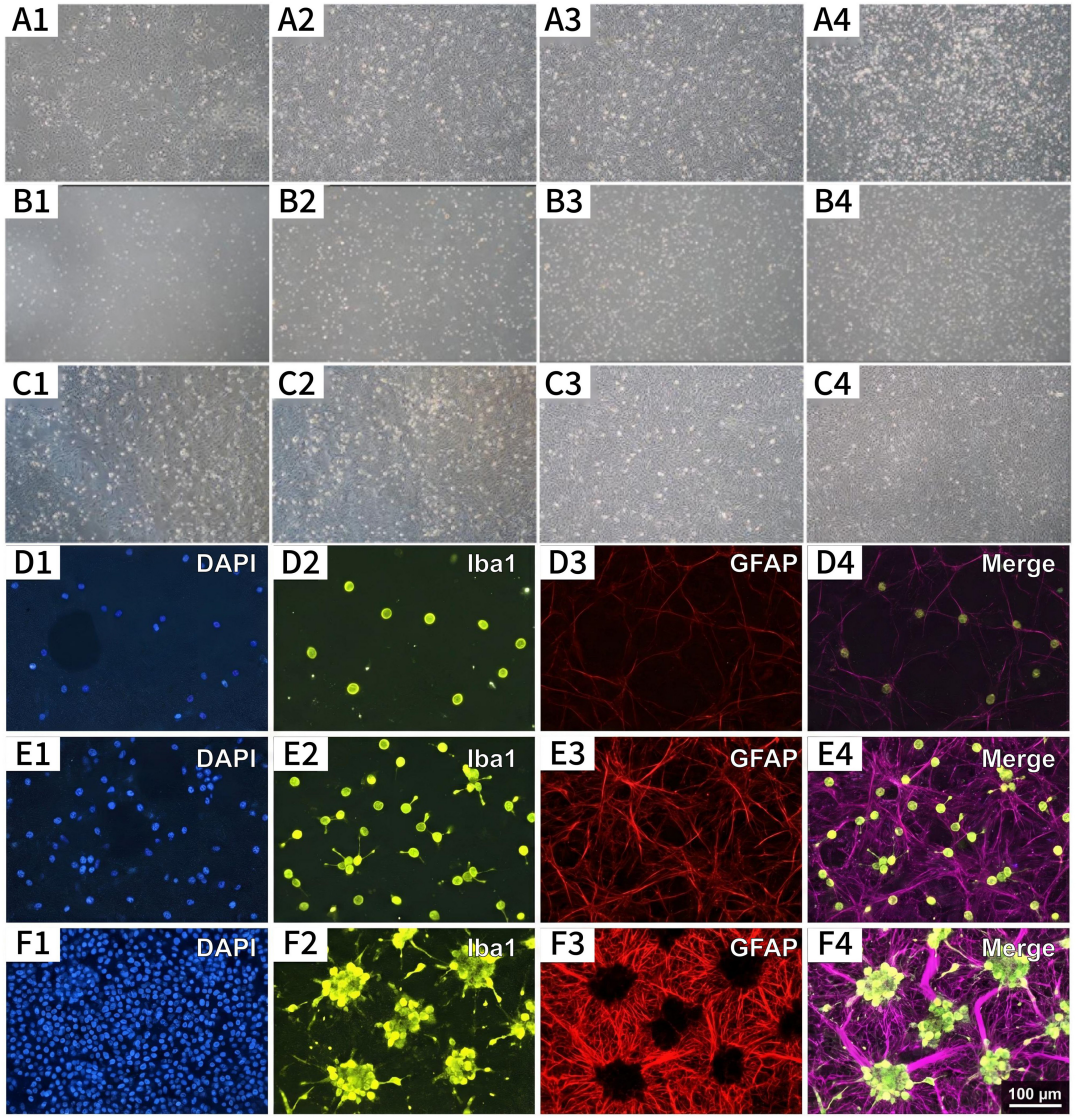
Morphological and immunofluorescence characterization of mixed glial cultures at different time points. **(A–C)** Phase-contrast microscopy: **(A)** Mixed glial cell culture prior to shaking separation. **(B)** Microglia collected after shaking separation, re-seeded and purified for 5 days. **(C)** Residual astrocytes after shaking separation, post-passage Day 5. **(D–F)** Dual-label immunofluorescence staining (Iba1, green; GFAP, red; DAPI, blue) of mixed glial cultures at Day 7 **(D)**, Day 10 **(E)**, and Day 14 **(F)** prior to shaking separation. All images at 100× magnification. Scale bar = 100 μm.

In mixed glial cell culture systems, microglia are situated above the astrocyte layer; however, the optimal time point for their separation has not been established. To determine the optimal separation window, oscillatory separation was systematically performed and compared on culture days 7, 10, 14, and 17. At each time point, following separation and purification, cells were immediately harvested for immunofluorescence analysis. The results indicated that the duration of culture significantly influenced the efficacy of cell separation (Figure 5).

**FIGURE 6:**
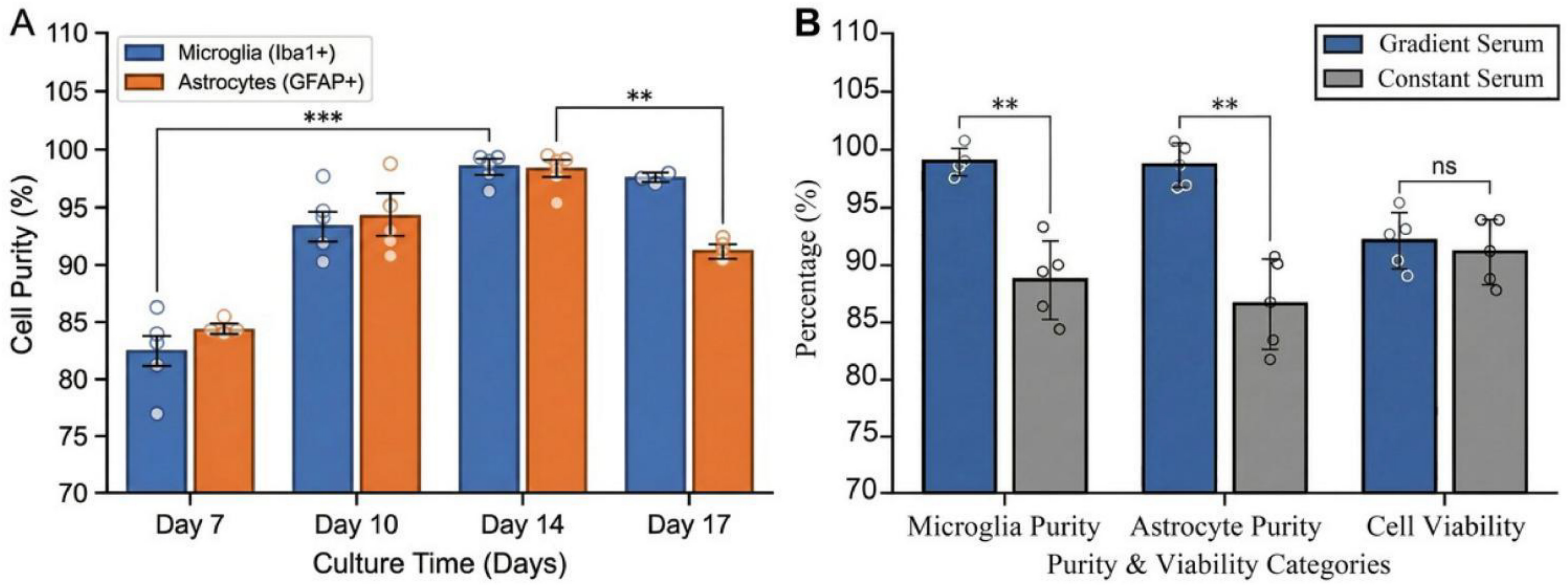
Validation of optimized separation time window and gradient serum strategy. **(A)** Comparison of microglial and astrocyte purity on the day of oscillatory separation (Day 14) at the indicated time points. Data are presented as mean ± SD (*n* = 5). Statistical analysis: one-way ANOVA with Tukey’s multiple comparisons test. **(B)** Purity of microglia and astrocytes evaluated on the day of separation (Day 14) under the optimized gradient serum protocol. Individual data points are shown. **P < 0.01, ***P < 0.001 (one-way ANOVA with Tukey’s multiple comparisons test).

To rapidly assess separation efficiency at different time points, samples were immediately collected after oscillatory separation for immunofluorescence analysis (Figure 6). On day 7 of culture, the stratified structure of the co-culture system was not yet mature, and the morphology and state of microglia had not fully stabilized. The Iba1 positivity rate on the day of separation was only 82.3% ± 3.5%, while the GFAP positivity rate for astrocytes was 84.5% ± 0.5%, indicating low purity. By day 10 of culture, a preliminary cellular stratification structure had formed. The Iba1-positive rate for microglia increased to 93.5% ± 2.8%, and the GFAP-positive rate for astrocytes reached 94.2% ± 3.1%. However, fibroblast contamination remained significant. By day 14, purity significantly improved following oscillatory separation: the Iba1-positive rate for microglia reached 98.6% ± 1.1%, and the GFAP-positive rate for astrocytes reached 98.4% ± 1.7%. By day 17 of culture, astrocytes exhibited excessive confluence with overly tight intercellular junctions. Following oscillatory separation, purity decreased to 97.7% ± 0.3% (microglia) and 91.3% ± 0.7% (astrocytes), with some cells sustaining damage under mechanical stress. Statistical analysis revealed that the purity on the day of oscillatory separation at the 14-day time point (microglia 98.6% ± 1.1%, astrocytes 98.4% ± 1.7%) were significantly higher than those at day 7 (82.3% ± 3.5% and 84.5% ± 0.5%, *P* < 0.001, Tukey’s *post-hoc* test after one-way ANOVA) and also significantly higher than the astrocyte purity at day 17 (91.3% ± 0.7%, *P* < 0.01). These results validate day 14 as the optimal isolation time window (Figure 6A).

To further characterize the cellular composition at each time point, dual-label immunofluorescence staining for Iba1 (green) and GFAP (red) was performed on mixed glial cultures prior to shaking separation (Figures 5D–F). At Day 7 (Figure 5D), GFAP^+^ astrocytes formed a sparse basal layer, with scattered Iba1^+^ microglia of predominantly round morphology loosely attached to the surface. By Day 10 (Figure 5E), the astrocytic monolayer became more confluent, and Iba1^+^ microglia increased in number and began to exhibit ramified morphology. By Day 14 (Figure 5F), a clearly stratified architecture was evident: a dense GFAP^+^ astrocyte bed overlaid by abundant Iba1^+^ microglia displaying fully ramified morphology, confirming that the co-culture system had reached structural maturity optimal for shaking separation.

To evaluate the efficiency of oscillatory separation at different time points, samples were collected immediately after shaking for immunofluorescence analysis (Figure 6). On day 7 of culture, the Iba1^+^ rate was 82.3% ± 3.5%, and the GFAP^+^ rate was 84.5% ± 0.5%. By day 10, the microglial Iba1^+^ rate increased to 93.5% ± 2.8%, and the astrocytic GFAP^+^ rate reached 94.2% ± 3.1%. By day 14, following oscillatory separation, the microglial Iba1^+^ rate was 98.6% ± 1.1%, and the astrocytic GFAP^+^ rate was 98.4% ± 1.7%. By day 17, purity decreased to 97.7% ± 0.3% for microglia and 91.3% ± 0.7% for astrocytes. Statistical analysis confirmed that the purity at the 14-day time point was significantly higher than that at day 7 (*P* < 0.001) and that astrocyte purity at day 14 was also significantly higher than at day 17 (*P* < 0.01) (Figure 6A).

### 3.3 Morphology and identification of microglia

Following a 14-days mixed glial culture, microglia were isolated by orbital shaking and collected. Phase-contrast microscopy and quantitative analysis revealed that microglial density increased linearly with culture time. On the day of isolation (day 0), the cell density was (2.1 ± 0.4) × 104 cells/cm^2^ (Figure 4A). At this initial time point, the cells had just undergone centrifugation and reseeding, and adhesion was not yet fully stabilized. By culture day 3, the density increased to (3.6 ± 0.5) × 104 cells/cm^2^ (Figure 4B), representing a ∼71% increase from day 0. Morphological quantification revealed a progressive transition from amoeboid to ramified phenotype during purification culture (Figures 4D–F). On Day 0, microglia exhibited a mean cell body area of 173.5 ± 32.8 μm^2^, high circularity (0.847 ± 0.058), and few primary processes (0.8 ± 0.4 per cell), consistent with a contracted, post-isolation state. By Day 3, cell body area increased to 287.4 ± 45.6 μm^2^ (*P* < 0.01 vs. Day 0), circularity decreased to 0.623 ± 0.087 (*P* < 0.001 vs. Day 0), and primary process number increased to 2.7 ± 0.6 (*P* < 0.001 vs. Day 0), indicating active process extension. By Day 5, microglia displayed a fully ramified morphology with markedly extended processes (Figure 4C), with cell body area further increased to 418.2 ± 68.3 μm^2^ (*P* < 0.001 vs. Day 0; *P* < 0.01 vs. Day 3), circularity decreased to 0.438 ± 0.095 (*P* < 0.001 vs. Day 0; *P* < 0.01 vs. Day 3), and primary process number reached 4.3 ± 0.8 (*P* < 0.001 vs. Day 0; *P* < 0.01 vs. Day 3). The relatively large standard deviation at Day 5 reflects the morphological heterogeneity inherent to primary microglial cultures, in which approximately 15%–20% of cells retained a round/amoeboid phenotype.

Dual-label immunofluorescence staining for Iba1 (green) and GFAP (red) was performed on Day 5 of purification culture to verify microglial identity and assess cross-contamination (Figures 3A–D). The vast majority of cells displayed strong Iba1 positivity with no detectable GFAP signal, confirming their microglial identity. Single-label quantification yielded an Iba1^+^ rate of 98.98% ± 1.21% on Day 5, slightly higher than the rate at the time of isolation (98.6% ± 1.1%, Section “3.2 Determination of the optimal separation time window”), indicating that the purification culture step effectively removed residual contaminating cells. Dual-label quantification further revealed that 97.36% ± 1.42% of cells were Iba1^+^/GFAP^–^, while GFAP^+^ contaminants (Iba1^–^/GFAP^+^) constituted only 0.78% ± 0.53%, and dual-positive cells (Iba1^+^/GFAP^+^) accounted for 0.52% ± 0.38%. These results confirm the high purity and minimal astrocytic cross-contamination of the purified microglial population.

### 3.4 Morphology and identification results of astrocytes

Following the removal of microglia via orbital shaking, the adherent cells remaining in the original culture flask were further cultured. Astrocytes exhibited characteristic morphological changes over time (Figures 3E–H). On culture day 1 (Figure 3E), the cells exhibited spindle-shaped or irregular morphologies and were relatively dispersed. By day 3 (Figure 3F), the cellular processes had extended and begun to interconnect. By day 5 (Figure 3G), the cells had formed a dense monolayer with a characteristic “pavement-like” morphology. Abundant processes were interwoven into a network, and contaminating cells were markedly reduced.

Quantitative analysis of astrocyte spreading confirmed the progressive increase in monolayer coverage (Figure 3H). The area fraction was 28.6% ± 5.3% on Day 1, reflecting the early post-passage re-attachment phase. By Day 3, coverage increased significantly to 62.4% ± 7.8% (*P* < 0.001 vs. Day 1), as astrocytes entered a phase of rapid proliferation and process extension. By Day 5, the area fraction reached 87.3% ± 4.6% (*P* < 0.001 vs. Day 1 and Day 3), forming a near-confluent monolayer consistent with the “pavement-like” morphology described above. The deceleration of growth between Day 3–5 (24.9 percentage point increase) compared to Day 1–3 (33.8 percentage point increase) is consistent with density-dependent contact inhibition characteristic of astrocytes.

Dual-label immunofluorescence staining for GFAP (red) and Iba1 (green) was performed on Day 5 post-passage (Figures 3I–L). Astrocytes displayed strong GFAP-positive fibrous networks with no detectable Iba1 signal, confirming their identity. Single-label quantification showed a GFAP^+^ rate of 98.81% ± 2.38%, slightly higher than the rate at the time of isolation (98.4% ± 1.7%, Section “3.2 Determination of the optimal separation time window”). Dual-label quantification demonstrated that 97.83% ± 1.95% of cells were GFAP^+^/Iba1^–^ (pure astrocytes), while residual Iba1^+^/GFAP^–^ microglia constituted only 0.36% ± 0.28%, and dual-positive cells accounted for 0.24% ± 0.19%. These results confirm that the passage purification and cross-pattern shaking steps yielded a highly pure astrocyte population with negligible microglial contamination.

### 3.5 Cell survival rate detection results

To evaluate the impact of key operational steps on cell viability, viability was assessed by trypan blue exclusion at five time points (Table 2). Viability after papain digestion (T0) was 94.32% ± 2.15%, indicating that the digestion conditions were well tolerated. Following differential adhesion (T1), viability was 93.87% ± 1.96%, showing no significant difference compared to T0 (*P* > 0.05). After oscillatory separation (T2), trypan blue viability decreased to 92.45% ± 2.34%, representing the lowest value across all time points (*P* < 0.05 vs. T0). After 5 days of purified culture (T3), viability increased to 95.67% ± 1.82% (*P* < 0.05 vs. T2). On day 3 after passaging (T4), astrocyte viability was 96.23% ± 1.45% (*P* < 0.05 vs. T2), with no significant difference between T3 and T4 (*P* > 0.05).

**TABLE 2:** Cell viability assessed by trypan blue exclusion and CCK-8 metabolic assay at each procedural stage.

Time point	Description	Trypan blue viability (%)	CCK-8 viability (%)	Difference (pp)
T0	After papain digestion	94.32 ± 2.15	90.56 ± 3.27	3.76
T1	After differential adhesion	93.87 ± 1.96	89.14 ± 2.89	4.73
T2	After shaking separation on day	92.45 ± 2.34*	88.23 ± 3.41**	4.22
T3	Day 5 of microglial culture	95.67 ± 1.82	92.43 ± 2.56	3.24
T4	Day 3 after astrocyte passage	96.23 ± 1.45	94.18 ± 1.87	2.05

Data are expressed as mean ± SD (*n* = 5 independent experiments).

*P < 0.05,

**P < 0.01 vs. T0 (one-way ANOVA with Tukey’s *post-hoc* test). pp, percentage points.

CCK-8 metabolic viability assays performed at the same time points showed a concordant trend (Table 2, Supplementary Figure 1). CCK-8 values were systematically lower than trypan blue values by 2.1–4.7 percentage points across all time points, consistent with the higher sensitivity of metabolic assays to sub-lethal cellular stress. The lowest CCK-8 viability was also observed at T2 (88.23% ± 3.41%; *P* < 0.01 vs. T0), confirming that oscillatory separation constitutes the most stressful step in the protocol. Both assays demonstrated full viability recovery by T3 and T4 (CCK-8: 92.43% ± 2.56% and 94.18% ± 1.87%, respectively), indicating that cells regained metabolic competence during purification culture. The concordance between membrane-integrity and metabolic-activity assessments provides complementary evidence that all procedural steps maintain cell viability within acceptable limits.

### 3.6 Flow cytometric validation of cell purity

To provide an independent, large-scale quantitative assessment of cell purity complementary to immunofluorescence-based evaluation, multicolor flow cytometry was performed on purified microglia (Day 5 of purification culture) and astrocytes (Day 3 post-passage) (Supplementary Figures 2A–C).

The gating strategy is illustrated in Supplementary Figure 2A. Sequential gating on FSC-A/SSC-A (debris exclusion), FSC-H/FSC-A (doublet discrimination), and Zombie NIR negativity (live cell selection) yielded viable cell fractions of 89.3% ± 3.2% for microglial preparations and 92.7% ± 2.1% for astrocyte preparations. The slightly lower viability in microglia preparations may reflect the greater mechanical stress imposed by the preceding orbital shaking separation step.

In the microglial population (Supplementary Figure 2B), CD11b^+^ cells constituted 97.12% ± 1.58% of live-gated events. Further resolution of CD45 expression intensity revealed that the predominant population was CD11b^+^/CD45low (95.37% ± 1.84%), consistent with the canonical homeostatic microglial immunophenotype, whereas a minor CD11b^+^/CD45high fraction (1.75% ± 0.92%) likely represents residual peripheral macrophage contamination. CD11b^–^ cells accounted for only 2.88% ± 1.58% of the total live population.

In the astrocyte population (Supplementary Figure 2C), ACSA-2^+^ cells constituted 94.65% ± 2.73% of live-gated events. Within the ACSA-2^–^ fraction (5.35% ± 2.73%), CD11b^+^ cells represented only 0.82% ± 0.45%, indicating minimal microglial carryover. The remaining ACSA-2^–^/CD11b^–^ cells (4.53% ± 2.41%) may include astrocyte subpopulations with low surface expression of ATP1B2 (the antigen recognized by ACSA-2), as well as a small number of undifferentiated progenitor cells.

The flow cytometry-derived microglial purity (CD11b^+^: 97.12% ± 1.58%) was marginally lower than the immunofluorescence-based estimate (Iba1^+^: 98.98% ± 1.21%), a discrepancy of 1.86 percentage points. This minor difference is methodologically expected, as flow cytometry interrogates substantially larger cell numbers (∼20,000 vs. 300–500 per sample) and the enzymatic dissociation required for single-cell suspension preparation may transiently compromise surface CD11b epitope integrity. For astrocytes, the difference between ACSA-2^+^ flow cytometric purity (94.65% ± 2.73%) and GFAP^+^ immunofluorescence purity (98.81% ± 2.38%) was more pronounced (4.16 percentage points), attributable to the inherent difference between a surface marker (ACSA-2/ATP1B2) and an intracellular cytoskeletal protein (GFAP), which do not necessarily co-label identical cell populations. Collectively, flow cytometric analysis at the scale of >20,000 events per sample corroborated the high purity of both glial populations established by immunofluorescence.

### 3.7 Fibroblast exclusion verification

Given that meningeal-derived fibroblasts constitute the primary source of contamination in primary glial cultures, immunofluorescence staining for the reticular fibroblast marker ER-TR7 was performed to directly verify the efficacy of the three-stage purification strategy (Supplementary Figure 3). NIH 3T3 mouse embryonic fibroblasts, serving as a positive control, displayed intense ER-TR7 immunoreactivity (red) with a diffuse cytoplasmic and extracellular matrix distribution pattern, yielding a positivity rate of 96.84% ± 1.37% (Supplementary Figure 3A). As expected, no GFAP or Iba1 signal (green channel) was detected in NIH 3T3 cells.

In purified astrocyte cultures (Supplementary Figure 3B), no unequivocal ER-TR7-positive cells were identified across all fields examined. Rigorous quantification across five independent experiments (2,847 DAPI^+^ cells counted in total) revealed that only 4 cells exhibited faint ER-TR7-like fluorescence, yielding a nominal positivity rate of 0.14% ± 0.13%. Critically, the fluorescence intensity of these cells was approximately 8%–12% of the mean fluorescence intensity of NIH 3T3 positive controls, and all four cells co-expressed GFAP in the green channel. This signal intensity, approaching the secondary-antibody-only background level (0.08% ± 0.06%), is consistent with non-specific background fluorescence rather than genuine fibroblast contamination. The GFAP^+^ rate in these preparations was 98.52% ± 1.86%, consistent with the values reported in Section “3.4 Morphology and identification results of astrocytes.”

In purified microglial cultures (Supplementary Figure 3C), no ER-TR7-positive cells were detected (0.00% ± 0.00%; 2,536 DAPI^+^ cells counted across five experiments). The Iba1^+^ rate was 98.74% ± 1.33%, consistent with the data reported in Section “3.3 Morphology and identification of microglia.” These results provide direct molecular evidence that the combination of differential adhesion, gradient serum reduction, and modified orbital shaking effectively eliminates fibroblast contamination to below the detection threshold. The complete absence of ER-TR7 signal in microglial cultures and the negligible background-level signal in astrocyte cultures rule out the possibility that fibroblasts survived the purification procedure by adopting a glial-like morphology.

## 4 Discussion

### 4.1 Selection of donor age and establishment of a mild digestion system

Donor age is a critical determinant of cell yield, purity, and culture success rate. While primary glial cell cultures are commonly established from newborn rats within the postnatal day 0 to 3 (P0-P3) window, significant variations exist across this age range in terms of dissection difficulty, cell proliferation capacity, and the risk of contamination by non-glial cell types. In this study, these factors were systematically compared, leading to the identification of postnatal day 2 (P2) as the optimal harvest time point. Glial cells harvested at P2 are in an active proliferative state and yield significantly higher cell densities compared to those harvested at P0. Compared to rats at P4 or beyond, P2 rats have less mineralized cranial bones and a more loosely structured pia mater and vascular network. These anatomical characteristics facilitate the precise dissection of target tissue under a stereomicroscope. Complete removal of the pia mater is crucial for ensuring culture purity (Gingras et al., 2007). Residual meningeal fibroblasts are the primary source of contamination in primary glial cultures, as they proliferate at a significantly faster rate than glial cells (McCarthy and de Vellis, 1980). Under standard culture conditions, these fibroblasts can rapidly overgrow the glial cells. Therefore, meticulous dissection during tissue harvest is essential to prevent non-glial cell contamination at the source, thereby establishing a critical foundation for the success of subsequent purification steps. Cell suspensions prepared following meticulous removal of the dura mater, and subsequently subjected to differential adhesion and seeding, demonstrate low levels of fibroblast contamination during subsequent culture. This results in favorable starting conditions for the subsequent steps of graded serum reduction and orbital shaking separation.

The optimization of enzymatic digestion parameters represents a critical determinant of final cell quality and viability. Conventional trypsin-based digestion protocols can be overly aggressive, resulting in non-specific proteolysis of cell surface receptors and potential loss of functional epitopes (Huang et al., 2010). In microglia, enzymatic damage leading to the loss of key surface markers (e.g., CD11b, CX3CR1) not only impedes accurate immunophenotypic identification but can also disrupt receptor-mediated signaling pathways, thereby altering cellular responses. For astrocytes, enzymatic over-digestion can compromise the structural integrity of critical proteins such as aquaporin 4 (AQP4) and glial fibrillary acidic protein (GFAP), leading to aberrant cytoskeletal organization and consequent morphological alterations (Nikodemova and Watters, 2012). Therefore, papain was chosen as the digestive enzyme for this protocol. Papain, a cysteine protease with broad substrate specificity and moderate proteolytic kinetics, is well-suited for isolating sensitive neural cell populations (Brewer et al., 1993). An optimized papain digestion protocol (0.25% w/v, 37 °C, 20 min) was established, which effectively dissociated neonatal rats brain tissue while preserving cell integrity. This optimized protocol yielded a post-digestion cell viability of 94.32% ± 2.15%, a value consistent with those achieved by other established mild digestion methods (Saura et al., 2003; Hu et al., 2022). Alternative enzymatic strategies, such as the time-gradient digestion approach described by Li et al. (2021), have also demonstrated the importance of precise control over digestion parameters for preserving cell heterogeneity and viability.

It should be noted, however, that enzymatic dissociation and subsequent *in vitro* culture inevitably alter the transcriptomic and functional profiles of primary glial cells relative to their *in vivo* counterparts. Recent single-cell RNA sequencing studies have demonstrated that isolated microglia rapidly undergo *ex vivo* transcriptomic activation, with upregulation of immediate early genes and inflammatory mediators within hours of extraction (Marsh et al., 2022). Similarly, astrocytes maintained in serum-containing media acquire a reactive phenotype characterized by elevated expression of proliferative and inflammatory gene modules that diverge substantially from their quiescent *in situ* state (Cahoy et al., 2008). Cadiz et al. (2022) further demonstrated that the choice of dissociation enzyme, culture substrate, and media composition collectively shape the resulting transcriptomic landscape, underscoring the importance of standardized protocols for cross-study comparability. While the present protocol was optimized to maximize cell yield, viability, and purity, the phenotypic fidelity of the resulting cultures to *in vivo* glial populations was not assessed and constitutes an inherent limitation of all serum-based primary culture systems.

The rational selection of culture substrates is also critical for optimal cell adhesion and the maintenance of cellular morphology. This study systematically compared the performance of two coating materials, poly-L-lysine (PLL) and poly-D-lysine (PDL). The results showed that cells cultured on PDL achieved a significantly higher adherent cell density (245.8 ± 38.4 vs. 152.6 ± 41.7 cells/mm^2^; *P* = 0.0038; Figure 1C), representing an approximately 61% increase, with lower inter-field variability (CV: 15.6% vs. 27.3%). Furthermore, astrocytes exhibited fully extended processes and a characteristic multipolar morphology. This difference can be attributed to the superior resistance of the D-lysine isomer to degradation by cell-secreted matrix metalloproteinases, which allows the PDL coating to provide durable electrostatic adhesion support. Throughout the 10- to 14-days culture period, the PDL coating remained intact. This stability provided the essential microenvironmental support necessary for maintaining near-physiological cellular morphology.

### 4.2 Development and mechanistic analysis of a three-stage purification strategy

Although preconditioning optimization can significantly improve initial cell population quality, meningeal-derived fibroblasts may still compromise the purity of target cells in later culture stages due to their inherent proliferative advantage (Abbasi et al., 2020). Therefore, a three-step tandem purification system was established, comprising differential adhesion, gradient serum screening, and modified oscillatory separation, with each step designed to address specific purification challenges. The differential adhesion step exploits inherent differences in adhesion kinetics between cell types. Fibroblasts express high levels of integrins α5β1 and αvβ3, as well as fibronectin receptors, leading to significantly faster adhesion (approximately 50% within 15 min). In contrast, neuroglial cells express lower levels of adhesion molecules and adhere more slowly (approximately 50% within 30–45 min) (Agalave et al., 2020). This temporal window was utilized to remove a portion of rapidly adhering non-glial cells during initial plating. Although quantitative analysis of cell composition before and after this step was not conducted, no significant fibroblast overgrowth was observed in subsequent culture, indicating its utility in reducing non-target cell contamination.

The gradient serum reduction strategy constitutes the core methodological innovation of this study. Control experiments validated the efficacy of this approach. Compared to cultures maintained in constant 10% FBS, the gradient serum regimen yielded an increase in purity of 8.4% for microglia and 9.9% for astrocytes (*P* < 0.01), without compromising cell viability (*P* > 0.05). These results indicate that periodic nutritional stress selectively suppresses fibroblasts, which typically exhibit high metabolic demands, while preserving the physiological activity of glial cells. The established gradient serum protocol (10% ^→^ 5% ^→^ 2% ^→^ 10% FBS) creates a cyclical low-nutrient environment. This stress induces cell cycle arrest specifically in metabolically active fibroblasts. Under low-serum conditions (2% FBS), fibroblasts undergo G0/G1 phase arrest as a consequence of unmet metabolic demands (Rodrigues et al., 2023). Prolonged nutrient deprivation can further trigger apoptotic pathways (Dalman et al., 2010). In contrast, astrocytes exhibit greater resilience to low-serum stress. They adapt through metabolic plasticity and by utilizing glycogen stores, mechanisms that involve the upregulation of glucose transporters and activation of glycolytic pathways (Prah et al., 2019). Critically, this approach obviates the need for antimitotic agents such as cytarabine (Ara-C), thereby entirely avoiding the potential hidden toxicity that such chemical compounds can exert on glial cell mitochondria and metabolic networks.

Optimized oscillation-based separation constitutes a critical step for the precise isolation of both cell types. This protocol refines the classical oscillatory separation method established by McCarthy and de Vellis (1980), which exploits differential cell adhesion strength. Specifically, microglia, often described as highly motile surveillant cells, exhibit weaker adhesion, whereas astrocytes, which provide structural and metabolic support, adhere more tenaciously (Jolivel et al., 2015). Parameter optimization identified 200 rpm for 12 h as the optimal condition. Following oscillatory separation, cell viability was maintained at 92.45% ± 2.34%. This value is significantly higher than the 85%–90% viability typically reported for traditional methods (Tamashiro et al., 2012). A supplementary manual “cross-shaking” step was introduced after supernatant removal. This step utilizes transient hydrodynamic force to further eliminate residual oligodendrocyte precursor cells and cellular debris (Zheng et al., 2025), contributing to the final stable purity of over 98%. The efficacy of this three-stage purification strategy in eliminating fibroblast contamination was directly confirmed by ER-TR7 immunofluorescence staining (Section “3.7 Fibroblast exclusion verification”; Supplementary Figure 3). No ER-TR7-positive fibroblasts were detected in microglial preparations (0.00%), and only negligible background-level signal was observed in astrocyte preparations (0.14%, with fluorescence intensity at 8%–12% of the NIH 3T3 positive control threshold). These results provide molecular-level evidence that the combined action of differential adhesion, gradient serum starvation, and orbital shaking effectively eliminates meningeal fibroblasts, corroborating previous reports that serum deprivation selectively induces G0/G1 arrest and apoptosis in fibroblasts while sparing glial populations (Rodrigues et al., 2023; Dalman et al., 2010).

Cell viability exhibited significant recovery during post-purification culture, as demonstrated by both membrane-integrity and metabolic assessments (Trypan blue-T3: 95.67% ± 1.82%, T4: 96.23% ± 1.45%; CCK-8 – T3: 92.43% ± 2.56%, T4: 94.18% ± 1.87%), indicating the restoration of population homeostasis. The systematic difference between the two assays (2.1–4.7 percentage points, with CCK-8 consistently lower) is methodologically informative: trypan blue exclusion provides a binary assessment of membrane physical integrity, whereas CCK-8 measures mitochondrial dehydrogenase activity, which is sensitive to sub-lethal metabolic perturbation. Early apoptotic or metabolically stressed cells may retain membrane integrity (trypan blue–negative) while exhibiting reduced mitochondrial function (lower CCK-8 signal). The convergence of the two measurements at later time points (T4 difference: 2.05 pp) further supports the conclusion that cells achieve full metabolic recovery during purification culture. This recovery can be attributed to several factors: the clearance of isolation-generated debris and apoptotic cells through medium exchanges; the resolution of acute isolation stress within approximately 72 h; and the establishment of a stabilized microenvironment via autocrine/paracrine signaling within the purified population (Jolivel et al., 2015; Stansley et al., 2012). The high post-isolation viability ensures preserved cellular physiological functions, thereby providing a reliable foundation for subsequent functional investigations.

### 4.3 Methodological comparisons, limitations, and directions for improvement

In this study, highly purified microglia and astrocytes were obtained, as confirmed by a multi-method validation strategy encompassing single-label immunofluorescence, dual-label immunofluorescence, and multicolor flow cytometry. Microglial purity was consistently high across all three methods: Iba1^+^ single-label (98.98% ± 1.21%), Iba1^+^/GFAP^–^ dual-label (97.36% ± 1.42%), and CD11b^+^ flow cytometry (97.12% ± 1.58%), yielding an inter-method coefficient of variation of 1.05%. Astrocyte purity showed slightly greater methodological variability: GFAP^+^ single-label (98.81% ± 2.38%), GFAP^+^/Iba1^–^ dual-label (97.83% ± 1.95%), and ACSA-2^+^ flow cytometry (94.65% ± 2.73%), with an inter-method CV of 2.20%. The lower ACSA-2-based estimate is attributable to the inherent difference between a surface antigen (ACSA-2/ATP1B2) and an intracellular cytoskeletal protein (GFAP); not all GFAP^+^ astrocytes express high levels of surface ATP1B2, particularly following enzymatic dissociation that may transiently compromise surface epitope integrity (Kantzer et al., 2017). Dual-label immunofluorescence further demonstrated minimal cross-contamination, with dual-positive cells (Iba1^+^/GFAP^+^) constituting <1% in both populations (microglia: 0.52%; astrocytes: 0.24%). This purity level significantly exceeds that achieved by traditional shaking separation methods (90%–95%) and is comparable to results from magnetic-activated cell sorting (MACS) and flow cytometric sorting (FACS) (Zelenka et al., 2022; Pan and Wan, 2020). Although MACS and FACS can achieve purities of ≥99%, the present method offers distinct advantages: it incurs lower per-separation costs; employs a gentle, antibody-free separation process that avoids potential receptor-mediated activation (Bohlen et al., 2019); enables simultaneous isolation of both glial types from a single P2 neonatal rat, reducing animal usage by 50%; and requires only basic cell culture equipment. Several recently published protocols warrant specific comparison. Mishra and Raju (2020) described a simplified shaking-based isolation achieving microglial purities of 92%–95% with a shorter culture period (7–10 days); however, their protocol did not incorporate gradient serum reduction or fibroblast exclusion verification, and the reported purity was based solely on single-marker immunofluorescence without flow cytometric corroboration. Woolf et al. (2025) introduced a Percoll density gradient centrifugation step following enzymatic dissociation, which effectively separates myelin debris and enriches glial progenitors from adult rodent brain tissue – an application domain where the present neonatal-tissue-based protocol is not directly applicable. Notably, Hamanaka et al. (2025) reported significant sex-dependent differences in microglial yield and cytokine secretion profiles from neonatal mouse cultures, with male-derived microglia exhibiting higher baseline TNF-α expression. The present study used mixed-sex P2 rat pups without sex stratification, which may introduce uncontrolled variability; future studies should consider sex as a biological variable in accordance with current reporting guidelines (Clayton and Collins, 2014).

The cell purity data demonstrated low variability, as evidenced by a standard deviation and coefficient of variation (CV) of approximately 1.0%–1.7%. This high reproducibility is attributed to stringent experimental controls: (1) standardized dissection protocols, including meticulous removal of the dura mater; (2) precisely controlled culture conditions, with temperature maintained at 37.0 °C ± 0.5 °C and a timed shaking regimen; and (3) a consistent data acquisition workflow using fixed thresholds in ImageJ, with 5–8 randomly selected fields (≥200 cells per field) analyzed per sample. Each sample underwent two independent cycles of immunofluorescence staining and imaging, and the mean of these replicates was used for statistical analysis (Schneider et al., 2012). Furthermore, all procedures during the method optimization phase were performed by a single experienced technician (>2 years in primary glial culture) to minimize operator-dependent variation.

This study has several limitations. First, the single-operator, single-center experimental design limits the assessment of the method’s generalizability. Key steps, including leptomeningeal dissection, differential adhesion time control, and cross-hand shaking, require substantial operational expertise, and reproducibility across different laboratories and operators requires further validation. Although gradient serum controls confirmed the strategy’s overall efficacy, the lack of a systematic factorial design precludes the determination of the independent contribution of each purification step. Consequently, the relative importance of factors such as PDL coating, differential adhesion, and optimized shaking parameters remains unclear; the high purity reported here should be interpreted as the result of multiple combined measures. All parameters were optimized for P2 newborn rats and would likely require adjustment for application to other ages, strains, or species (Gingras et al., 2007). The application of this protocol to adult neural tissues presents specific technical challenges, including high levels of myelin debris and robust cell–matrix adhesions, both of which can adversely affect cell yield and quality (Gingras et al., 2007). Although the present revision added quantitative substrate comparison data (Figure 1C), the analysis was limited to adherent cell density at a single time point (Day 3); comprehensive assessment of adhesion kinetics, cell spreading dynamics, and substrate degradation over the full 14-days culture period was not performed. Although the present revision substantially strengthened the purity validation framework through the addition of flow cytometry (CD11b/CD45/ACSA-2), dual-label immunofluorescence (Iba1/GFAP), and fibroblast exclusion staining (ER-TR7), phenotypic characterization remained limited to identity markers rather than functional subtype classification. Accumulating evidence underscores the inherent limitations of relying on single canonical markers for definitive glial cell identification: Iba1 (AIF1) is a pan-microglial marker that does not discriminate between homeostatic and disease-associated microglial states (Jurga et al., 2020); moreover, Iba1 is also expressed by infiltrating macrophages and other myeloid cells, limiting its specificity for identifying resident microglia (Uff et al., 2022). Furthermore, a recent study demonstrated that certain goat-derived anti-Iba1 antibodies exhibit off-target labeling of vasopressin neurons in the mouse hypothalamus, although this cross-reactivity was not observed with rabbit-derived antibodies or in rat tissue (Lichtenstein et al., 2026). The rabbit anti-Iba1 antibody (Abcam, ab178846) employed in the present study is therefore not subject to this specific limitation. Early studies highlighted that GFAP-based purity assessment of primary astrocyte cultures can substantially overestimate actual purity, as the confluent astrocyte monolayer visually obscures contaminating cells residing beneath or between GFAP^+^ cells (Saura, 2007). Similarly, GFAP labels primarily fibrous and reactive astrocyte subpopulations while underrepresenting protoplasmic astrocytes in certain brain regions (Khakh and Deneen, 2019), and a substantial proportion of astrocytes in serum-containing primary cultures are GFAP-negative (Xu, 2018). The adoption of more discriminative markers – such as TMEM119 and P2RY12 for homeostatic microglia (Bennett et al., 2016; Butovsky et al., 2014), CD45 intensity gating for distinguishing resident microglia from infiltrating macrophages, and Aldh1L1 or GLT-1 for broader astrocyte subtype coverage (Cahoy et al., 2008) – would provide a more comprehensive characterization of the isolated populations. Furthermore, single-cell RNA sequencing would enable unbiased assessment of population heterogeneity and the degree of *ex vivo* activation (Marsh et al., 2022). Furthermore, although the 10–14-days culture cycle is shorter than traditional methods, it may still be impractical for time-sensitive experiments. Potential effects of prolonged low-serum culture on cellular metabolism and gene expression also warrant further investigation. Future studies should evaluate inter-laboratory reproducibility through multi-center collaboration, quantify the contribution of individual purification steps using factorial designs, and optimize enzymatic digestion protocols for adult tissues to broaden the method’s applicability.

Furthermore, the morphological characterization performed in this study was confined to basic quantitative parameters–cell body area, circularity index, and primary process number–derived from phase-contrast images. Future investigations incorporating immunofluorescent labeling combined with confocal imaging at controlled low-density plating conditions would permit the application of more comprehensive morphometric analyses, including Sholl analysis and 3DMorph-based branching quantification, thereby enabling a more refined assessment of microglial activation states and morphological heterogeneity.

In summary, the glial cell isolation protocol described in this study provides a practical methodology for obtaining and analyzing highly purified populations of astrocytes and microglia from the cerebral cortex of postnatal day 2 (P2) rats. Specific parameters within this protocol can be modified to accommodate diverse downstream applications, thereby enhancing its adaptability for various experimental needs. Furthermore, the high cell viability and specific immunofluorescence identification confirmed in this study support the suitability of this protocol for subsequent functional investigations.

## 5 Conclusion

This study addressed the methodological bottleneck in primary glial cell isolation techniques–the difficulty in balancing purity and cost–by establishing a synchronized isolation protocol based on gradient serum concentration regulation and time window optimization. This protocol employs a three-tier purification system: differential adhesion, gradient serum screening, and modified oscillatory separation. A 10%–5%–2% FBS gradient strategy suppresses fibroblast proliferation, enabling simultaneous isolation of both glial cell types from a single P2 neonatal rat cerebral cortex within an optimal separation window (days 10–14, with day 14 being the peak time point). Following oscillatory separation, microglia exhibited an Iba1 positivity rate of 98.6% ± 1.1%, while astrocytes demonstrated a GFAP positivity rate of 98.4% ± 1.7%. After purification and culture, purity further increased to 98.98% ± 1.21% and 98.81% ± 2.38%, respectively. These results were independently validated by a multi-method strategy: dual-label immunofluorescence demonstrated that cross-contamination between microglia and astrocytes was <1% (Iba1^+^/GFAP^+^ dual-positive cells: 0.52% in microglia cultures and 0.24% in astrocyte cultures); multicolor flow cytometry confirmed microglial purity (CD11b^+^: 97.12% ± 1.58%) and astrocyte purity (ACSA-2^+^: 94.65% ± 2.73%) at the scale of >20,000 events per sample; and ER-TR7 immunofluorescence staining confirmed the complete absence of fibroblast contamination in both purified populations. Gradient serum control experiments confirmed that this strategy increased cell purity by 8.4%–9.9% compared to constant serum culture. Cell viability, assessed by both trypan blue exclusion and CCK-8 metabolic assay, remained above 92% following key procedural steps and recovered to over 95% post-purification culture, with the concordance between membrane-integrity and metabolic-activity assessments providing complementary evidence that all procedural steps maintain cell viability within acceptable limits. This method enables simultaneous isolation of both glial cell types from a single animal, reducing animal usage by 50% and aligning with the 3Rs principles of laboratory animal ethics. The three-tier purification system established in this study provides a systematic methodological framework for primary glial cell isolation, offering valuable reference for parameter optimization. However, its reproducibility across laboratories and operator variability require further validation.

## Data Availability

The original contributions presented in this study are included in the article/Supplementary material, further inquiries can be directed to the corresponding author.
