# Supplementary material for: Method for the simultaneous isolation of primary astrocytes and microglia from the neonatal rats cerebral cortex
Source: Front Cell Neurosci. 2026 Apr 21;20:1787397. doi: 10.3389/fncel.2026.1787397 (PMC13138937; doi:10.3389/fncel.2026.1787397)
Supplement: Supplementary file 1 [file Supplementary_file_1.docx]

**Supplementary Figure S1.** Cell viability assessed by trypan blue exclusion and CCK-8 metabolic assay at each procedural stage.


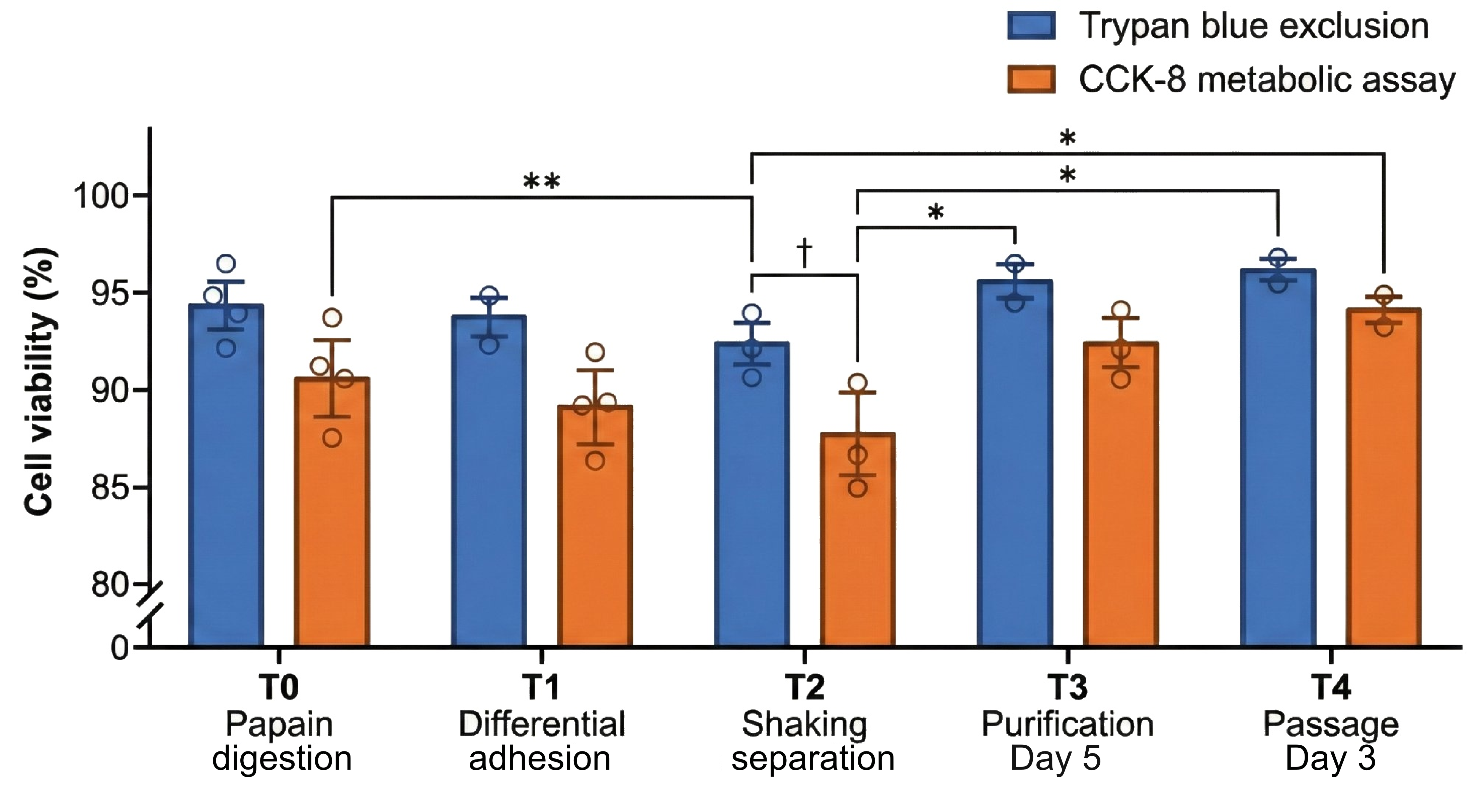


Data are presented as mean ± SD with individual data points (n = 5). *P < 0.05, **P < 0.01 vs. T0 (one-way ANOVA with Tukey's post-hoc test); †P < 0.05, paired-sample t-test comparing trypan blue and CCK-8 values at the same time point.

**Supplementary Figure S2.** Flow cytometric validation of purified glial cell populations.


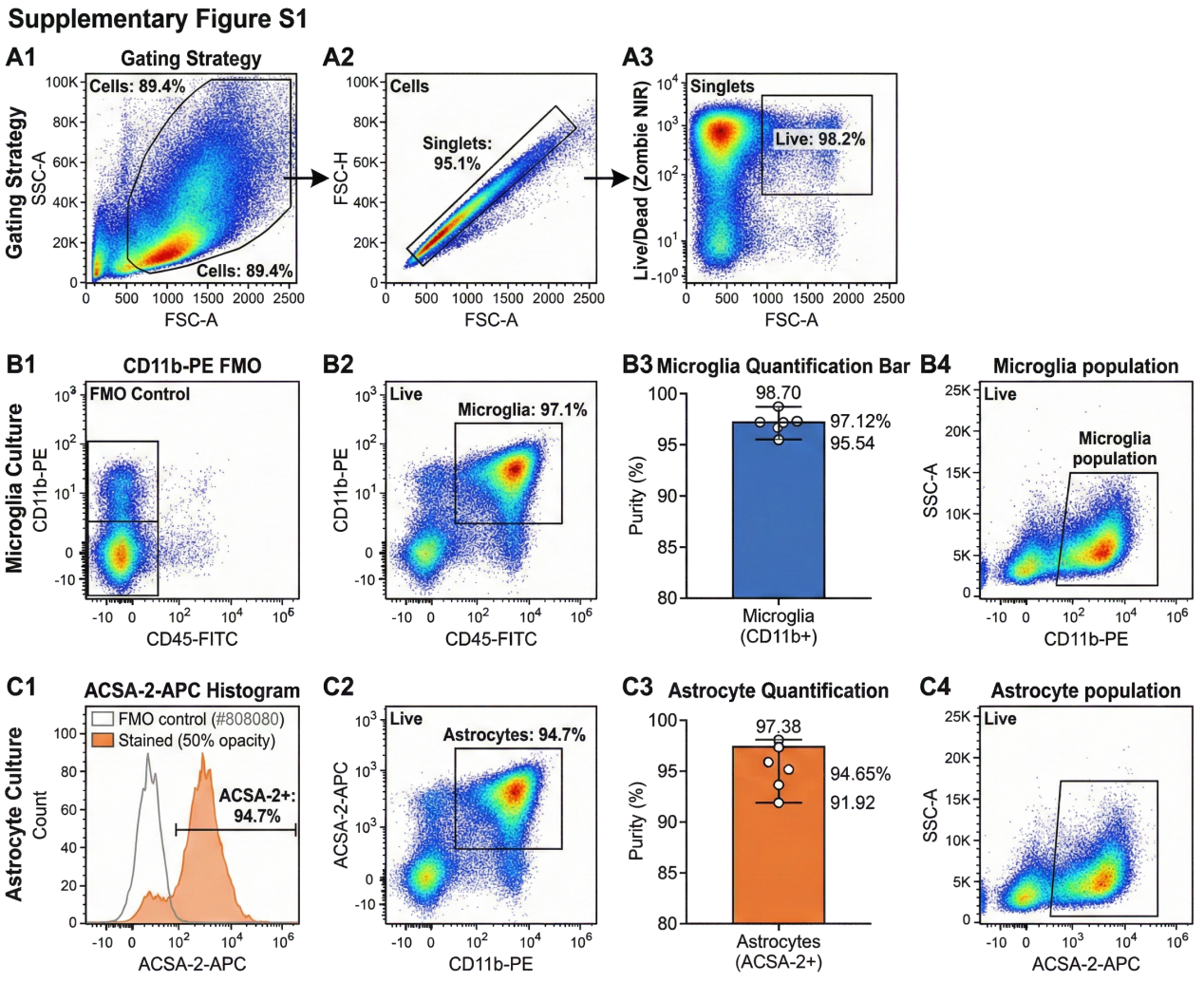


(A) Representative gating strategy: FSC-A/SSC-A scatter for debris exclusion, FSC-H/FSC-A for doublet discrimination, and Zombie NIR for live/dead discrimination. Fluorescence-minus-one (FMO) controls (gray) are overlaid to define positive thresholds. (B) Microglial purity analysis: left panel, CD11b expression histogram with FMO overlay; right panel, CD11b vs. CD45 density plot distinguishing CD11b⁺/CD45low microglia from CD11b⁺/CD45high macrophages. (C) Astrocyte purity analysis: ACSA-2 expression histogram with FMO overlay. Quantification bar graphs display mean ± SD with individual data points (n = 5).

**Supplementary Figure S3.** ER-TR7 immunofluorescence staining for fibroblast exclusion verification.


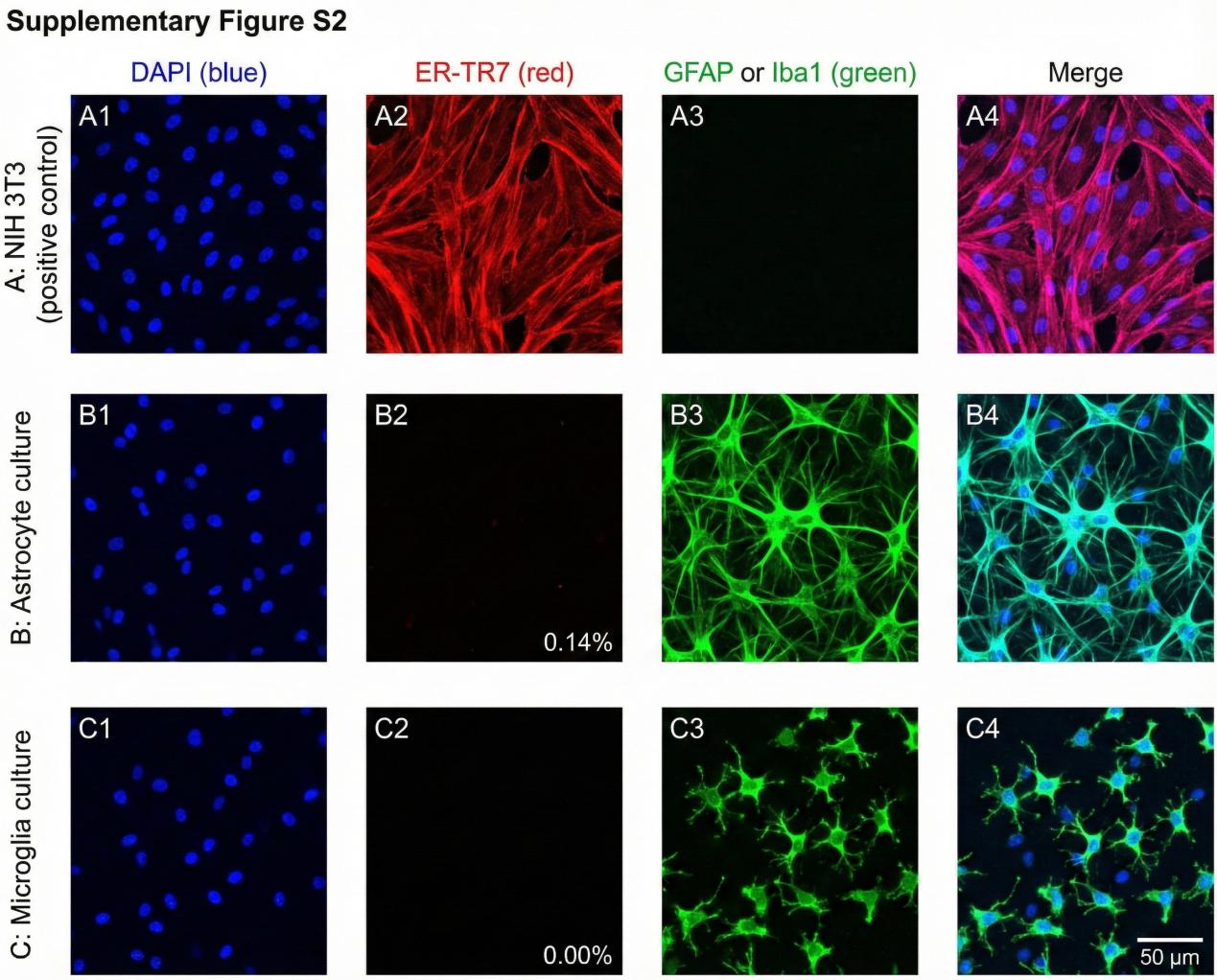


1. NIH 3T3 positive control: ER-TR7 (red), GFAP/Iba1 (green), DAPI (blue), and merged image. (B) Purified astrocyte culture: ER-TR7 (red), GFAP (green), DAPI (blue), and merged image. (C) Purified microglial culture: ER-TR7 (red), Iba1 (green), DAPI (blue), and merged image. Scale bar = 50 μm. Magnification: 200×.
